# Effective removal of heavy metal ions (Pb, Cu, and Cd) from contaminated water by limestone mine wastes

**DOI:** 10.1038/s41598-024-82861-2

**Published:** 2025-01-11

**Authors:** Aya T. Fathy, Mohamed A. Moneim, Ezzat A. Ahmed, Abdalla M. El-Ayaat, Fatma M. Dardir

**Affiliations:** Geology Department, Faculty of Science, Assiut University, Assiut, Egypt

**Keywords:** Limestone mining wastes, Calcium oxid, Adsorption, Heavy metals, Mechanism, Thermodynamic, Environmental chemistry, Environmental impact

## Abstract

Limestone mining waste and its derived CaO were checked as an adsorbents of pb^2+^, Cu^2+^, and Cd^2+^ ions from water solution. The characterization of Limestone and calcined limestone was studied by using X-ray diffraction (XRD), Fourier transform infrared spectroscopy (FTIR), thermogravimetric analysis (TGA), Scanning Electron Microscope (SEM), and Surface area measurements (BET). The optimum conditions of sorbent dosage, pH, initial concentration, and contact time factors were investigated for pristine limestone and calcined limestone absorbents. The results indicate that the optimum initial concentrations of (C_i_) were 1200, 500, and 300 ppm for Pb, Cu, and Cd, respectively, using calcined limestone adsorbent, while using the pristine limestone adsorbent, the corresponding optimum initial concentrations were 700, 110, and 50 ppm. In the ternary system sorption, the results indicated that the selectivity sequence of the studied metals by limestone can be expressed as Pb^2+^ > Cd^2+^ > Cu^2+^, while calcined limestone exhibits a higher selectivity for Pb^2+^ compared to Cu^2+^ and Cd^2+^. Hence, various adsorption isotherm and kinetic models were examined to explore different patterns and behaviors of adsorption. So, the results indicate that calcined limestone has great potential for eliminating cationic heavy metal species from industrial water solutions.

## Introduction

Water toxicity by hazardous substances such as heavy metals is a critical issue worldwide, necessitating that all harmful effluents be cured to reduce their harmful effects. Many industries, e.g., battery manufacturing, metal plating, mining, ceramic, glass, etc., are the main factors that increase the concentration of heavy metals in the environment and are accountable for heavy metal contamination^1–6^. Lead (Pb), cadmium (Cd), and copper (Cu) are examples of the category of seriously perilous heavy metals^7,8^. In addition, these metals in an aqueous solution may exist as free ions, soluble salts, ligand-associated ions, or ions bound to particulate matter^9^. The existence of elevated levels of these metals in the environment may provoke chronic health risks to humans and ecosystems ^10^. For instance, lead poisoning can have a deleterious impact on the central neural system^11^. Long-range exposure to cadmium complexes may trigger osteoporotic bones, kidney damage, and even death. Moreover, these complexes are foreseeable human carcinogens^12,13^. The effects of excess copper in the body are nausea, vomiting, plus eventual death^14^. Heavy metals can also contaminate soil and groundwater, which could harm plants and animals over the long run^15^. Therefore, the world is trending towards reliable, low-cost, and practical methods for wastewater treatment.

The traditional techniques for removing heavy metal ions from an aqueous solution are chemical precipitation^16–19^, nanofiltration^20,21^, ion exchange^22,23^, electrolytic treatment^24^, coagulation-flocculation^25^, and adsorption^26,27^. Among these technologies, adsorption is an effective method for purifying contaminated water, because of its User-simplicity, and Passivity^28^.

Adsorbents that are used in adsorption processes can be recycled and used again^29,30^.

In recent times, Various varieties of adsorbents have been investigated for removing heavy metals from water. For example, activated carbon^31–33^ is the most commonly utilized adsorbent, carbon nanotubes^34^, and metal–organic frameworks (MOFs)^35^ and zeolite minerals^27,36^, these adsorption agents have high surface areas; nevertheless, these adsorbents can be highly costly and arduous to prepare^37^. So, it’s essential to use adsorbents that are cheap, available, eco-friendly, and simple to prepare^28^.

Worth mentioning, there are many industrial, metallurgical, agricultural waste, and natural materials have been investigated as adsorbents for wastewater treatment^38,39^. For example, adsorbents that have been investigated are calcite^9,40^, sawdust^41^, metallurgical slags^42^, iron oxides^43^, siderite^44^, siderite/limestone^45^, activated phosphate^46,47^, zeolites^48^, and so on.

Among all the aforementioned adsorbents, adsorption using commercial activated carbon has been widely studied as an efficient adsorbent for heavy metal ions^49^. Commercial activated carbon illustrates significant adsorption ability in gas and liquid phases due to its high micropore volume, large specific surface area (SSA), favorable pore size distribution, thermal stability, and capability for rapid adsorption and low acid/base activity. Despite the advantages of the adsorption method, some disadvantages, including high preparation and recovery costs and the need for specialists, limit its use^50^. Thus, extensive research studies have been performed to find a low-cost alternative to commercial activated carbon with high adsorption capacity^51^.

The present work concerns the study of the utilization of Abu Tartur phosphate mining rock wastes. The values of these rock wastes must be considered in a feasibility study, particularly in the context of future continuous mining activities for phosphates. In this regard, it is worth mentioning that the Egypt Phosphate Company in the Abu Tartur sector has adopted a new and effective policy for the treatment of mining rock wastes. They classify and dump the wastes according to different rock types or grades.

The present work, therefore, aims to utilize the mining rock waste limestone from the Abu Tartur area to minimize environmental pollution and to address environmental problems such as the removal of heavy metals (Pb^2^⁺, Cu^2^⁺, Cd^2^⁺) from polluted water.

## Materials and methods

### Materials

All chemicals used obtained from commercial sources were of analytical grade. Merck product stocks of Pb^2**+**^, Cd^2**+**^, and Cu^2**+**^ standard solutions (1000 ppm) were used in the preparation of the contaminated water solution.

Limestone mining wastes were collected due to the activity Misr phosphate of company at Abu Tartur area, Egypt. calcined limestone (CaO) was prepared from limestone after ground and thermally decomposed at a temperature of 1100 °C for 2 h.

XRD, FT-IR, TGA, SEM, and BET Characterizations of the absorbents were carried out using the following instruments:

i. X-ray diffractometer with CuKα radiation generated at 40 kv and 30 mÅ. The powdered samples were scanned between 4° and 60° 2θ.
ii. Nicolet FT-IR spectrophotometer (model 6700) equipped with a data station.
iii. Thermal Analysis (DSC/TGA) was performed using the STA PT1600.
iv. SEM using JEOL Scanning Electron Microscope (Model: JSM-5400LV, Tokyo, Japan).
v. The specific surface areas of limestone and calcined limestone samples were measured using the Brunauer–Emmett–Teller (BET) method. The results were obtained by adsorption of nitrogen at 77 K using surface analysis (Micromeritics, USA, ASAP 2010). Before analysis, the samples were degassed under vacuum at 110 °C for 5 h.
vi. Pb^2+^, Cd^2+^, and Cu^2+^ concentrations analysis were determined using Atomic Absorption Spectrophotometer (AAS) Perkin Elmer. All investigations and analyses were carried out at Assiut University.

### Batch adsorption experiments

The batch method is frequently used to acquire data on removal effectiveness under equilibrium conditions^6^. A batch equilibrium experiment was carried out at room temperature using Pb, Cu, and Cd solutions in a mono and multi-metal sorption system in order to determine the efficiency of the pristine limestone and calcined limestone synthesized as adsorbents of heavy metals. The experiments were performed by adding a pre-determined amount of adsorbents (limestone or calcined limestone) to 25 mL of heavy metal-bearing solution with varying initial concentrations in 50 mL Erlenmeyer flasks. The flasks were placed in a rotary shaker at a constant speed of 100 rpm. At specific time intervals, samples were withdrawn out and centrifuged at 3500 rpm for 10 min. The supernatants were then filtered to ensure the solutions were free from adsorbent particles before measuring the residual heavy metal concentration. The amount of metal adsorbed and the percentage removal of heavy metals were calculated using the following Eqs. (1 and 2):

1 $${q}_{e}= ({C}_{i}-{C}_{e})\times V/m$$

2 $$Removal \%= ({C}_{i}-{C}_{e})\times 100/{C}_{i}$$

where q_e_ represents the amount adsorbed, while C_i_ and C_e_ respectively denote the initial and equilibrium concentrations of heavy metals (Pb^2+^, Cu^2+^, Cd^2+^). V is the solution volume, and m refers to the adsorbent weight.

Different solutions with different pH, contact time, initial concentration, and adsorbent dosage for each metal were used to evaluate the performance of the sorbents (Limestone and calcined limestone).

## Results and discussion

### Characterization

#### Crystallographic and chemical analysis

**Fig. 1:**
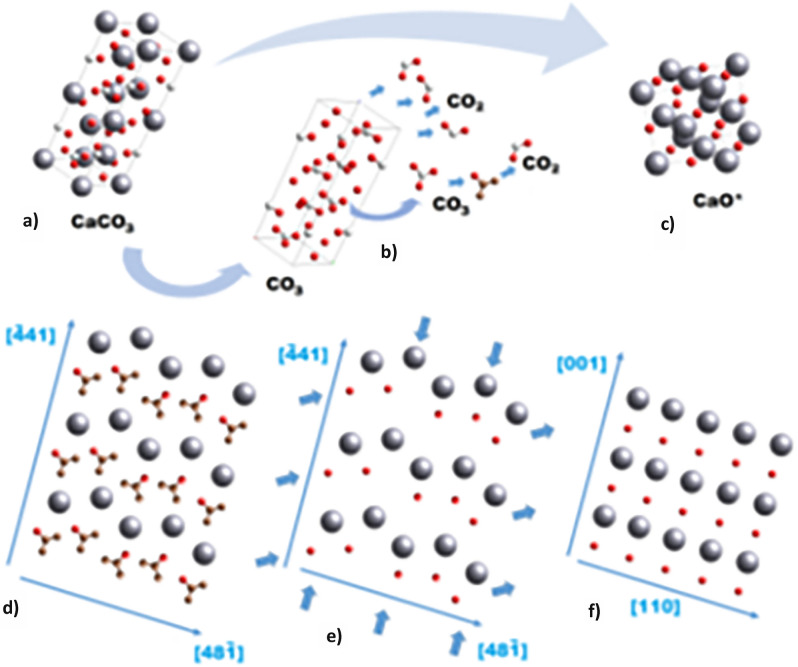
Schematizes the evolution from calcite to metastable CaO after calcination^52^.

Figure 1 shows the crystallographic unit cells of the three phases (CaCO_3_ calcite, CaO∗, and CaO lime) that would be expectedly involved in the calcination reaction^52^.

The limestone (CaCO_3_) was decomposed as the following Eq. (3)^53^:

3 $${\text{CaCO}}_{{{3}({\mathbf{s}})}} \to {\text{CaO}}_{{({\mathbf{s}})}} + {\text{CO}}_{{{2}({\mathbf{g}})}}$$

where s refers to solid and g refers to gas.

Major oxide analyses of calcined limestone (CaO) were carried out using os-ICP are given in Table 1. The results illustrated that after calcination to 1100 °C, CaO is the main component and represented by 99.74%, with subordinate amounts of MgO (0.2%) and FeO (0.06%).

**Table 1:** Chemical analyses of limestone samples after calcinated at 1100 °C.

Sample	CaO (%)	FeO (%)	MgO (%)
	99.736	0.064	0.2

Calcium oxide known as quicklime, is a white, crystalline solid. It is commonly used in various industrial processes, such as cement production^54^ and wastewater treatment.

#### X-ray diffraction (XRD)

**Fig. 2:**
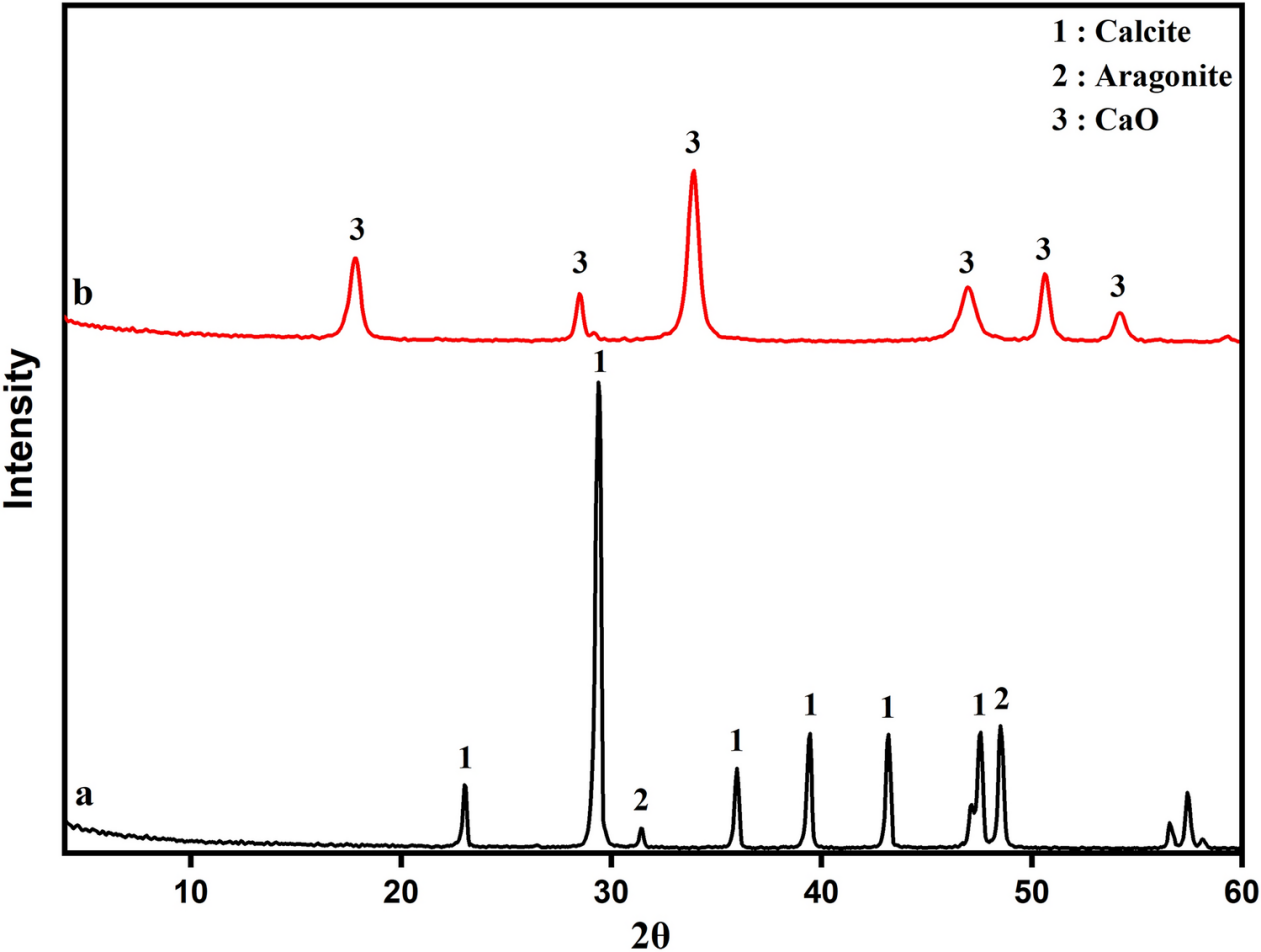
(**a**) XRD patterns of limestone (CaCO_3_) and (**b**) calcined limestone (CaO) (Following JCPDS card number 05–086 and 05–0453).

The results of the XRD diffractogram of limestone mining wastes and calcined samples (at T = 1100 °C) are shown in Fig. 2a,b. Based on the Joint Committee on Powder Diffraction Standards (JCPDS), the XRD analysis of limestone powder shows distinct reflections of the calcite crystals (CaCO_3_) phase at 2θ angles of 29.4°, 39.4°, and 43.1° ^55^ according to JCPS card number 05-086. Aragonite crystal phase is also identified at 2θ 31.2° based on JCPDS card number (05-0453) and 48.5° ^56,57^. Calcite is the main mineral phase composition of the limestone powder samples.

After calcining the limestone at 1100 °C for 2 h, the diffraction lines attributed to CaCO_3_ species disappear (Fig. 2b) due to the liberation of CO_2_, giving rise to a new diffraction line mainly located at 33.9° which is attributed to CaO (JCPDS card number 96-900-6716).

#### Fourier Transform Infrared Spectroscopy (FT-IR) analysis

**Fig. 3:**
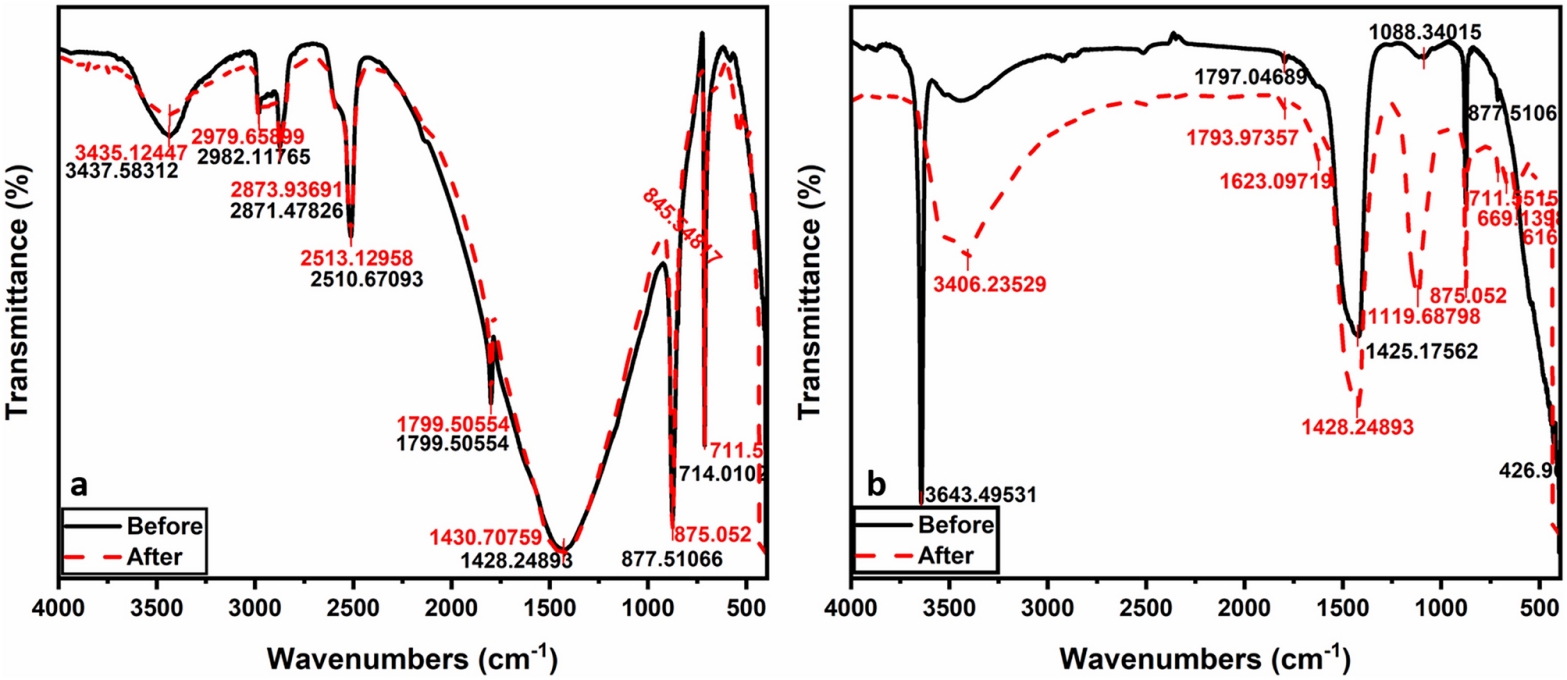
(**a**) FT-IR spectra of limestone(CaCO_3_) and (**b**) calcined limestone (CaO) before and after the sorption process.

The FTIR spectroscopy of powdered limestone (adsorbent) mining waste before the adsorption processes is presented in Table 2 and Fig. 3a. The carbonate phase presents with significant characteristics peaks at 1428 (ν_3_), 877 (ν_2_), and 714 cm^−1^ (ν_4_) which correspond to the carbonate group in CaCO_3_^58^. The smaller frequency bands at 2982, 2871, 2510, and 1799 cm^-1^ are attributed to the combination modes of different CO_3_^2-^ bands^59^. The details of these modes are summarized in Table 2. Bands at 3437 cm^-1^ correspond to H_2_O vapor.

**Table 2:** Infrared (IR) vibration modes of crushed limestone (L.st) and reference.

Observed vibrational frequencies (cm^-1^)			
Assignments	Present study	Reference^58^	Reference^59^
ν4—Symmetric CO_3_^2–^ deformation	714	711	712
ν2—Asymmetric CO_3_^2-^ deformation	877	875	874
ν3—Asymmetric CO_3_^2-^ deformation	1428	1425	1425
ν1 + ν4—CO_3_^2-^ deformation	1799	1799	1798
ν3—CO2 stretching mode	–	2362	–
2ν2 + ν4—CO_3_^2-^ deformation	2510	2515	2514
2ν3—CO_3_^2-^ deformation	2871	2873	2873
H_2_O stretching mode	–	3243	–

After the co-adsorption mechanism of heavy metal ions, the FTIR analysis showed that no chemical precipitation reactions occurred to produce new compounds after the limestone had adsorbed Cd^2+^ (100 ppm), Pb^2+^ (600 ppm) and Cu^2+^ (90 ppm) (Fig. 3a). This indicated that the reaction between limestone (CaCO_3_) is based only on physical adsorption^60^.

The FTIR spectroscopy results of the calcined limestone (adsorbent) powder after calcination at 1100 °C are given in Table 3 and Fig. 3b. According to Ref.^61^, the vibration band at 426 cm^-1^ was due to Ca–O symmetric vibration. Two frequency bands at 1425 and 1088 cm^-1^ are ascribed to C–O with respect to CO_2_ adsorbed on the surface of CaO^58^. Whereas, the sharp peak at 3643 cm^-1^ is characteristic of the OH group of Ca(OH)_2_. The peak height at 3643 corresponds to Ca(OH)_2_ concentration^62^.

**Table 3:** Infrared (IR) vibration modes of calcium oxide synthesized via calcination of L.st and references.

Assignments	Present study	Reference^58^	Reference^59^
ν Ca-O	426	–	424
ν C–O	1088	1063	866
ν C–O	1425	1441	1415
ν O–H	3643	3640	4643

Figure 3b shows the O-containing groups changed before and after the co-adsorption of heavy metal ions (Pb 600 ppm, Cu 150 ppm, and Cd 30 ppm). The O–H shifted from 3643 cm^-1^ before to 3406 cm^-1^ after adsorption. Asymmetric and symmetric peaks of COO- are observed at 1797 cm^-1^ to 1793 cm^-1^, and 1425 cm^-1^ to 1428 cm^-1^ respectively. A new peak appears at 1623 cm^-1^, which is due to the adsorption of Pb^2+^, Cu^2+^, and Cd^2+^ heavy metal ions. Shifting of O-containing peaks before and after heavy metal ions adsorption is presumably due to possible complexation and electrostatic interactions between Pb, Cd, and Cu ions and the O-containing groups^63^. In addition, representative peaks for Pb–O at 699 cm^-1^ and PbCO_3_ at 875 cm^-1^, as well as Cu–O at peaks 617 cm^-1^ and CuCO_3_ at 1119 cm^-1^, indicate that the heavy metal ions Pb, Cu, and Cd were adsorbed via ion exchange with the calcined limestone (CaCO_3_) adsorbent^64^.

Also, the noticeable shifts in the O–H and COO-bands in Fig. 3b indicate the coordination between OH or COOH groups and Cd^2+^. This proves that the metal exchange could be one of the mechanisms through which Cd^2+^ is adsorped onto calcined limestone^65^.

#### Thermogravimetric analysis

**Fig. 4:**
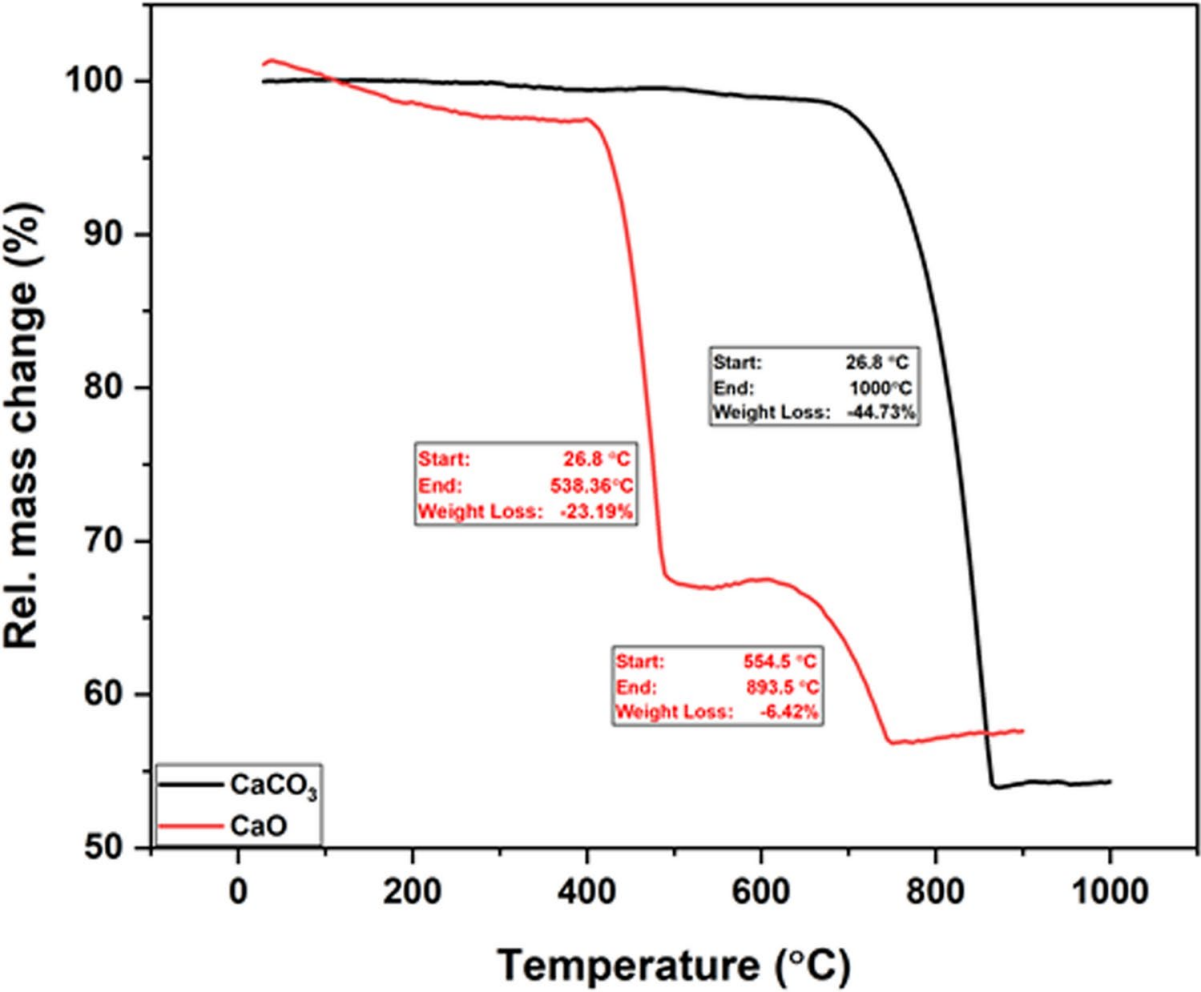
TGA of limestone (CaCO_3_) and calcined limestone (CaO), in an air atmosphere at a heating rate of 10 °C/min.

Thermogravimetric analysis (TGA) was carried out to determine the thermal stability of limestone (CaCO_3_) and calcined limestone (CaO), as well as their fractions of volatiles, by monitoring the weight loss as a sample was heated at a constant rate. TGA of limestone (CaCO_3_) (Fig. 4) shows the first weight loss was up to 2% due to water loss which occurred below 100 °C. The second weight loss of around 44% was due to the decomposition of CaCO_3_ to CaO and CO_2_^66^, which started from 760 to 860 °C. These results indicate that the CaO was expected to completely form after 860 °C. The purity of CaO detected from TGA was up to 54%.

While the TGA result of calcined limestone (CaO) revealed that below 100 °C the weight loss (3%) was due to water evaporation. The second weight loss up to 21% was started at temperatures up to 480 °C and happened due to evaporation of CO_2_^67^. The third weight loss up to 6% was at temperatures between 554 and 750 °C interpreted due to unreacted materials of the sample^67^. After passing the temperature around 750 °C, it appears that the mass change curve was relatively constant. The curve indicates that above temperatures of 750 °C, there is no change in the CaO compound^68^.

#### Scanning Electron Microscope (SEM)

**Fig. 5:**
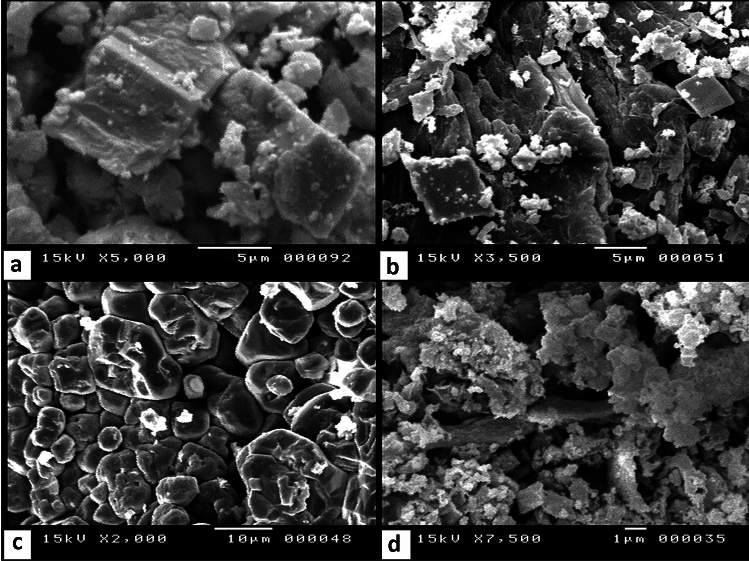
(**a**) and (**b**) SEM images of limestone (CaCO_3_) before and after the sorption process, (**c**) and (**d**) of calined limestone (CaO) before and after sorption respectively.

In order to understand the morphology and texture of natural limestone waste calcined limestone before and after heavy metals ions of Pb, Cu, and Cd adsorption, SEM analysis was investigated. SEM micrographs (Fig. 5a) of the limestone before adsorption show mainly the typical rhombohedral crystals of calcite carbonate with a size diameter of up to 5 µm. According to^69^, the carbonate precipitation is mainly calcite when the Ca^2+^:CO_3_^2-^ is 3:1 at a low rate of precipitation.

The SEM investigation of decomposed limestone to CaO is given in Fig. 5c. At 1100 °C, equidimensional, micrometer-sized CaO grains developed showing straight triple boundaries as well as neck contacts. These are typical features of a sintering process^53^.

After adsorption, the SEM microphotographs indicate only changes in sizes and forms (Fig. 5b,d) for limestone and calcined limestone, respectively.

Based on the FTIR results mentioned above, the adsorption of heavy metal ions (Pb^2+^, Cu^2+^, and Cd^2+^) is the process of ion exchange occurring mainly on the surfaces of the limestone and calcined limestone adsorbents.

#### Surface area analysis (BET)

**Fig. 6:**
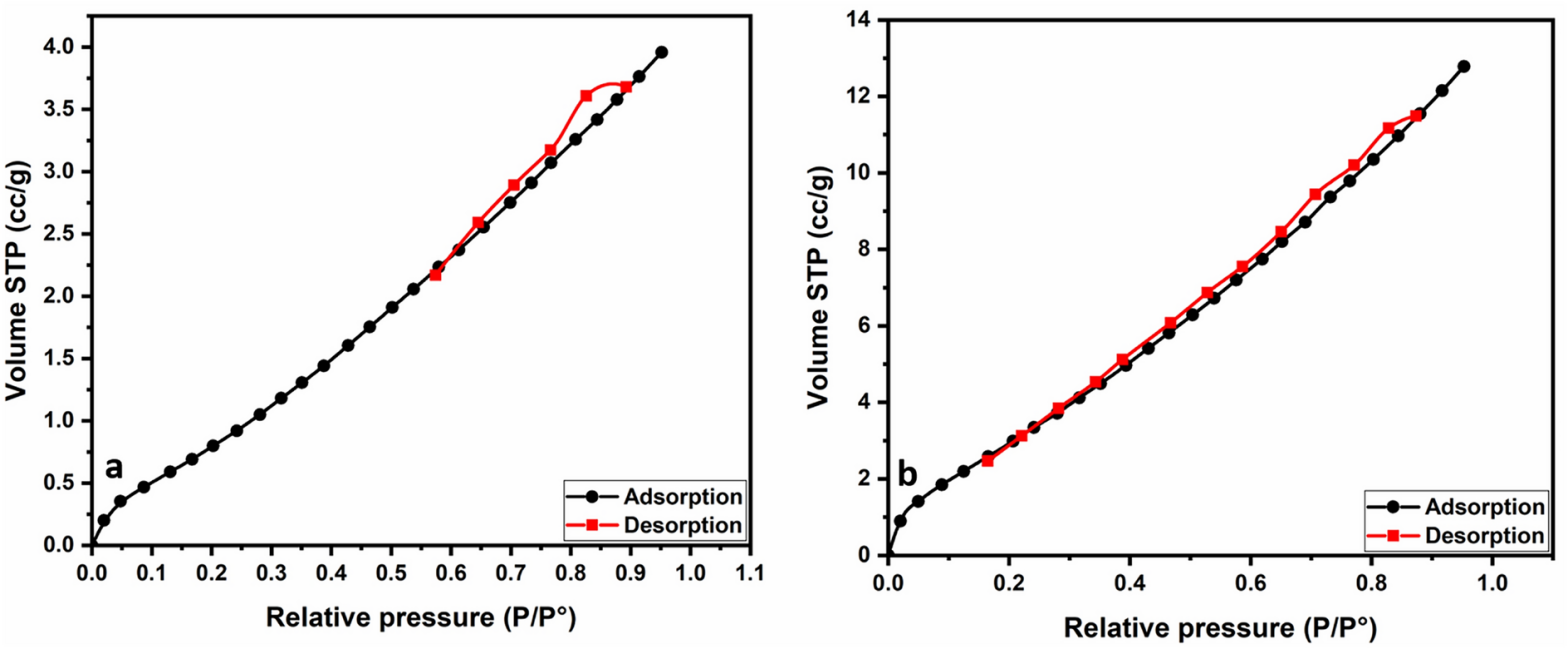
(**a**) Nitrogen adsorption–desorption isotherms of limestone (CaCO_3_) and (**b**) calcined limestone (CaO) samples.

The N_2_ adsorption–desorption isotherms for limestone (CaCO_3_) and calcined limestone are presented in Fig. 6a,b. Both materials exhibited a type IV isotherm^70,71^. The hysteresis loop of the type IV isotherms studied is classified as H_3_, indicating slit-shaped pores formed from the non-rigid aggregation of platy particles^72^. The BET surface areas of limestone and calcined limestone are measured to be 4.2 m^2^/g and 14.3 m^2^/g, respectively. Increasing the surface area after calcination because the surface become rougher and decreased in the size of particles due to CO_2_ emission. Thesr led to the surface area and the pore volume of the calcined limestone sample increased significantly^73,74^.

### Sorption results

#### *Optimum conditions of heavy metals (Pb*^*2*+^*, Cu*^*2*+^*, and Cd*^*2*+^*) removal*

##### Effect of adsorbent dosage

**Fig. 7:**
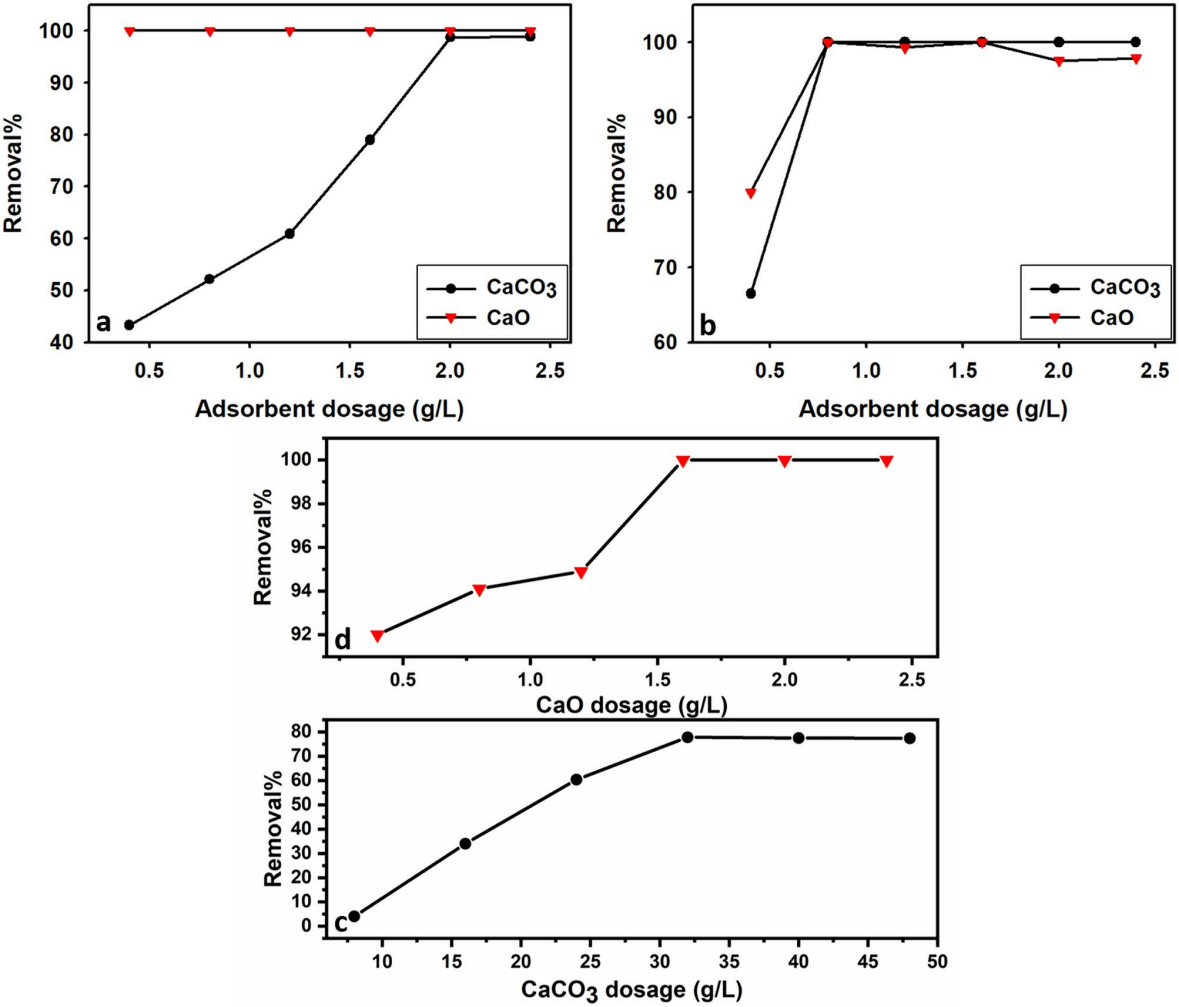
The relationship between adsorbent dosage and removal percentage of Pb^2+^ (**a**), Cu^2+^ (**b**), and Cd^2+^ (**c**, **d**) for limestone (CO_3_) and calcined limestone (CaO). Experiment conditions for CaCO_3_: t: 30 min., C_i_: pb^2+^  = 500 ppm, Cu^2+^ = 100 ppm, and Cd^2+^  = 50 ppm, pH: 5. Experiment conditions for CaO: t: 30 min., C_i_: pb^2+^  = 1000 ppm, Cu^2+^ = 500 ppm, and Cd^2+^  = 150 ppm, pH: 5.

The effect of limestone (CaCO_3_) adsorbent dosage on the removal of metal ions (Pb^2+^, Cu^2+^, Cd^2+^) was given in Fig. 7a–c. The dosages of limestone (CaCO_3_) adsorbent used in the batch experiments vary between 0.4 and 2.4 g/L for Pb and Cu, and 8–48 g/L for Cd, constant time 30 min, and initial concentrations 500 ppm for Pb, 100 ppm for Cu, and 50 ppm for Cd and 5 pH of the solution. The results indicate that the removal percentage of metal cations increased with increasing limestone dosage. The maximum removal efficiencies were 98.9% for Pb^2+^, 100% for Cu^2+^, and 77.8% for Cd^2+^ using adsorbent dosages of 2 g/L, 0.8 g/L, and 32 g/L, respectively. The process of sorption of heavy metals onto natural limestone begins with the dissolution of calcium carbonate, which results in the release of calcium into the solution^75^.

Figure 7a–d demonstrates how the removal percentage of metal ions (Pb^2+^, Cu^2+^, Cd^2+^) is influenced by calcined limestone adsorbent dosage. The removal of Pb ions reached 100% with a calcined limestone dosage equal to 0.4 g/L. The effect of removal is not affected by increasing the dosage from 0.8 to 2.4 g/L (Fig. 7a). The adsorbent dosage of 0.8 g/L and 1.6 g/L of calcined limestone were enough to remove 100% of Cu and 80% of Cd ions, respectively (Fig. 7b,d).

Increasing removal percent of Pb^2+^, Cu^2+^, and Cd^2+^ with increasing adsorbent dosages of limestone and calcined limestone may be due to the fact that the higher dosages of the adsorbent result in increased sorbent surface area and pore volume, thereby providing more capacity for adsorption^76,77^.

##### Effect of solution pH

**Fig. 8:**
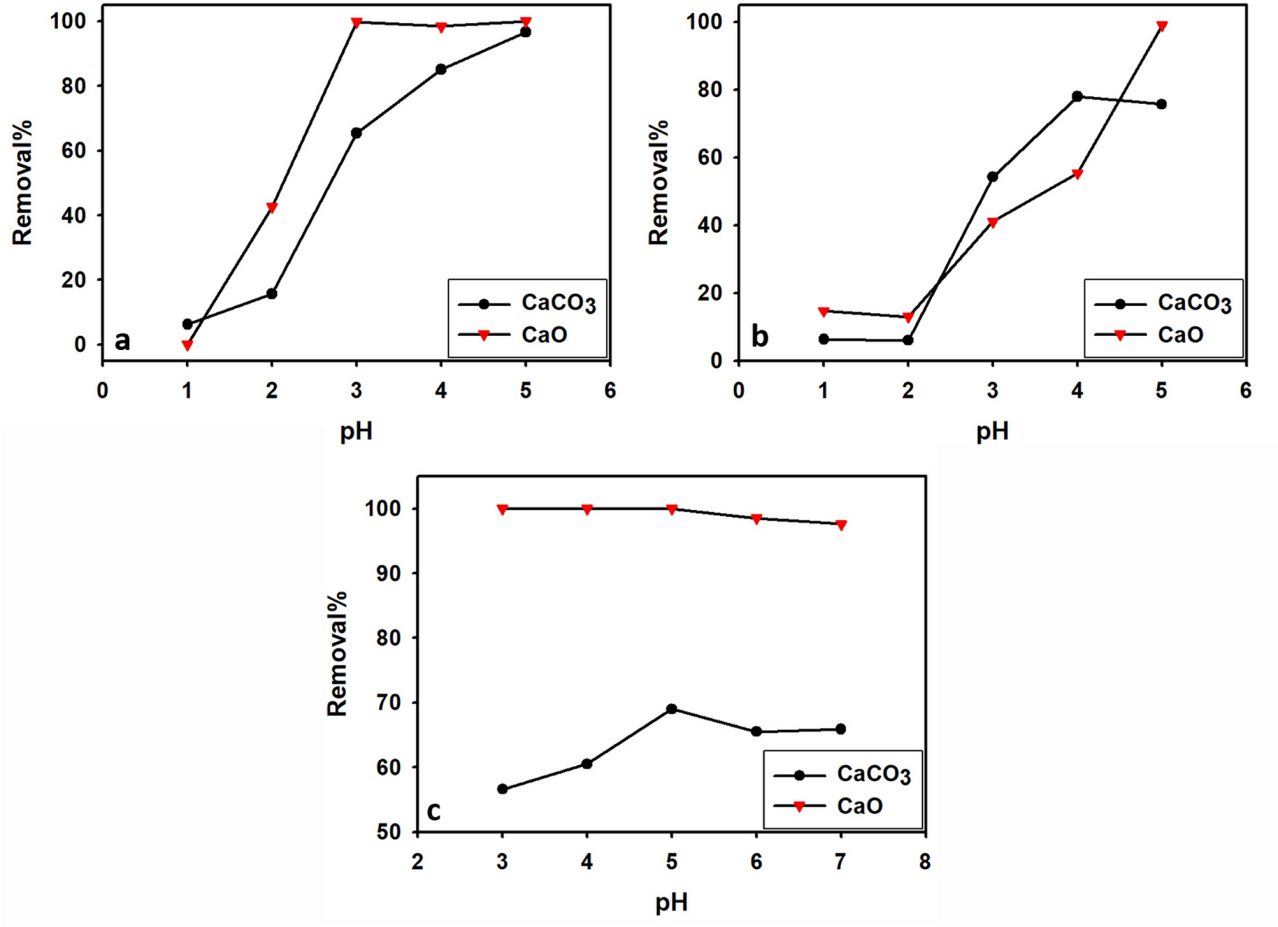
The relationship between solution pH and removal percentage of Pb^2+^ (**a**), Cu^2+^ (**b**), and Cd^2+^ (**c**) for limestone (CaCO_3_) and calcined limestone (CaO) adsorbent.

The effect of pH on the adsorption of Pb, Cu, and Cd, onto limestone and calcined limestone is presented in Fig. 8a–c. The adsorption process can be significantly influenced by pH, making it one of the most crucial factors to consider. The adsorption behavior of an adsorbent at different pH may be ascribed to several parameters, including its adsorption capacity, surface charges, and active sites^78^.

To prevent the precipitation of lead and copper ions as insoluble Pb(OH)^+^ and Cu(OH)_2_, the pH range of solutions is adjusted to a lower range, typically between 1.0 and 5.0, since at pH above 6.0, the concentration of hydroxide ions (OH^-^) increases and Pb^2+^ and Cu^2+^ ions react with hydroxide ions, causing them to precipitate out of solution^79–81^. The pH range in which Cd^2+^ ions remain in solution is between 3.0 and 7.0. Beyond a pH of 7.0, Cd^2+^ ions tend to precipitate out of the solution^82^.

Using limestone adsorbent, maximum removal efficiency reached 96.6% for Pb, 75.7% for Cu, and 69% for Cd at 5. In the case of calcined limestone adsorbent, the removal efficiency of Pb, Cu, and Cd reached 100% at pH values 5,5, and 3, respectively. Therefore, the adsorption process was significantly influenced by the pH of the aqueous solution, indicating its crucial role as a controlling parameter^83^.

#### *pH of zero point charge (pH*_*zpc*_*)*

The point zero charge (pzc) is described as the pH at which the net charge of the particles on the surface of adsorbent is zero. Similar to Bakatala et al.^84^ method, the salt addition method was used to determine the zpc of limesstone and calcined limestome adsorbents. Two sets of 50 mL centrifuge tubes (5 in each set) for each adsorbent with 0.05 gm of limestone for the first set, and 0.01 gm of calcined limestone in the other set. 25 mL of distalled was added to each tube.Then pH of each mixture were adjusted between 2 and 12 for each set using 0.1 M NaOH and 0.1 M HCl. After determination the intian pH for every tube, the solution was shaken for 1 h and the pH final measured.

**Fig. 9:**
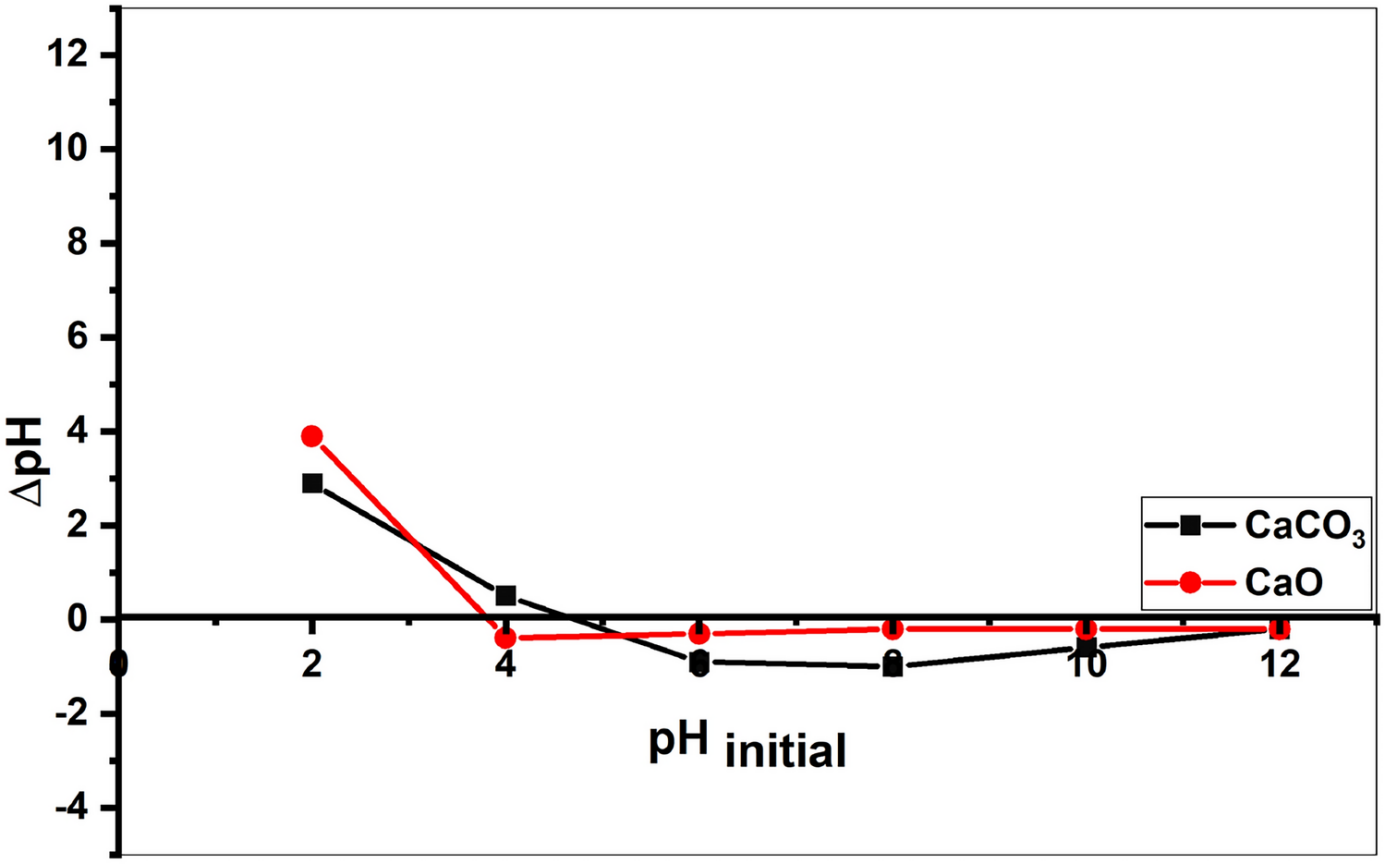
Zero-point-of-charge (pH_ZPC_) for limestone CaCO_3_ and calcined limestone CaO adsorbents.

The point of zero charge for limestone and calcined limestone was determined from a graph plotting the initial solution pH against The change in pH (∆pH = pH_final_ − pH_initial_), as shown in Fig. 9. The acidic and basic characteristics of the mineral surfaces are reflected in the value of the pH zero point charge (pH_zpc_). It is a significant property that defines the surface electrical neutrality of the particle^85^. The surface charge is negative when pH > pH_zpc_, which favors cation adsorption, and positive at pH < pH_zpc_^86,87^. It was evident that when ∆pH was zero, the pH_xpc_ values were 4.6 for limestone and 3.8 for calcined limestone adsorbent.. The increased adsorption of heavy metals was attributed to the electrostatic attraction between the negatively charged surface of the adsorbent and the electropositive heavy metal ions (Pb^2+^, Cu^2+^, Cd^2+^).

##### Effect of contact time

**Fig. 10:**
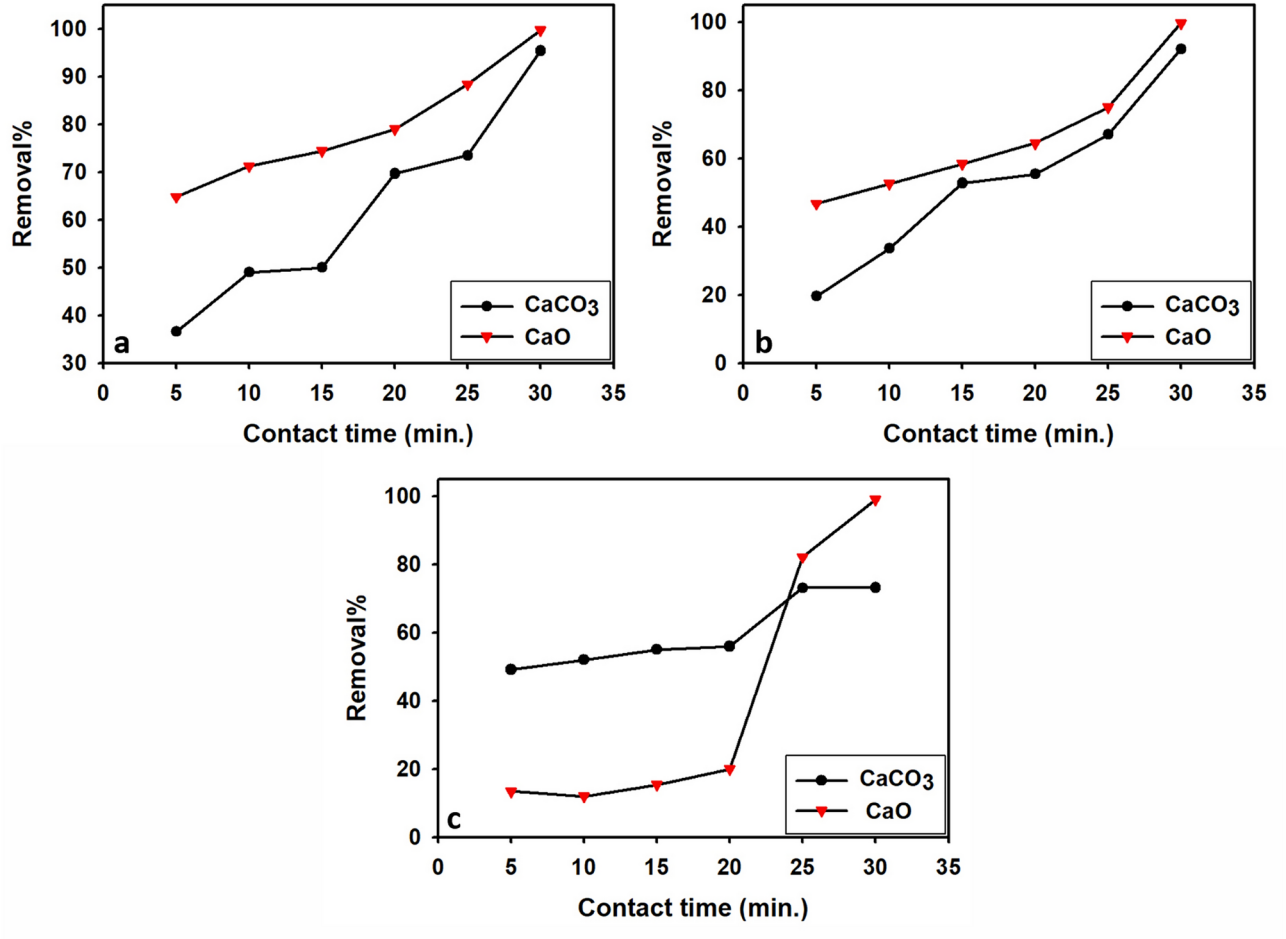
Influence of contact time on removal percentage of Pb^2+^ (a), Cu^2+^ (**b**), and Cd^2+^ (**c**) for limestone (CaCO_3_) and calcined limestone CaO adsorbents.

One of the important parameters that affect the adsorption process is contact time^88^. Figure 10a–c illustrates the impact of contact time on the adsorption processes of Pb, Cu, and Cd onto limestone and calcined limestone adsorbents. The adsorption efficiency was examined at a contact time range between 5 and 30 min. The results show that the highest efficiencies were achieved at different contact times for different metals and adsorbents.

In the case of limestone, the maximum efficiency for Pb was 95.4% at 30 min, for Cu it was 92.1% at 30 min, and for Cd, it was 73.1% at 25 min.

On the other hand, when using calcined limestone as an adsorbent, the optimum contact time to achieve maximum removal of Pb^2+^, Cu^2+^, and Cd^2+^ ions was 30 min, with a removal efficiency of 99.5% for all three metals.

##### Effect of initial concentration (C_i_)

The initial concentration of metals holds significant importance as it serves as a driving force to overcome the mass transfer resistance of molecules between aqueous and solid^89^.

**Fig. 11:**
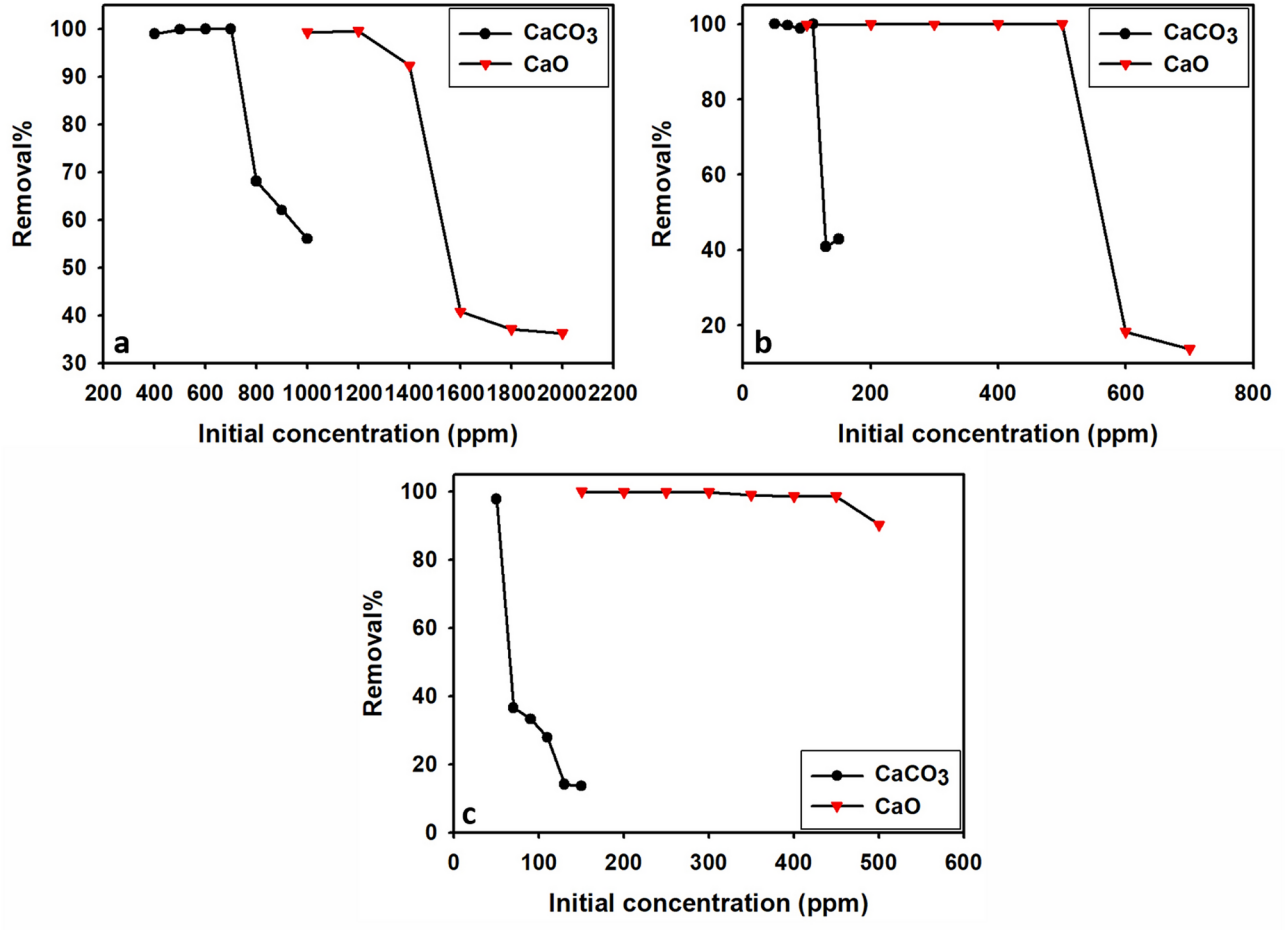
The effect of initial concentration (**a**) for Pb^2+^, (**b**) for Cu^2+^, and (**c**) for Cd^2+^ onto limestone (CaCO_3_) and calcined calcined limestone (CaO) adsorbents.

The effect of initial concentration on the adsorption of Pb, Cu, and Cd ions onto limestone (CaCO_3_) and calcined limestone adsorbents is given in Fig. 11a–c and Table 4. It was observed that the removal percentage of Pb, Cu, and Cd is almost nearly 100% with the initial concentration ranges 400–700 ppm for Pb, 50–110 ppm for Cu, and 50 ppm for Cd, using limestone as the adsorbent, at 5 pH and contact time 30 min. When using calcined limestone adsorbent under the same conditions of pH and contact time, the removal percentage reached 100% at initial concentrations of Pb ranging from 1000 to 1200 ppm, Cu ranging from 100 to 500 ppm, and Cd ranging from 150 to 300 ppm.

**Table 4:** The adsorption capacities (q_e_) of the heavy metals for CaCO_3_ and CaO.

CaCO_3_			CaO		
q_e_ (mg/g)			q_e_ (mg/g)		
Pb^+2^	Cu^+2^	Cd^+2^	Pb^+2^	Cu^+2^	Cd^+2^
230	62.5	1.5	2482.1	249.5	375
300	87.25	0.6	2988.1	500	499.6
350	112.375	0.9	3234.3	1000	624.2
272.5	137.5	0.6	1630.5	1250	748.8
276.75	66.5	0.6	1672.7	274.5	996.3
280.2	80.625	0.64	1259.3	243	1125

At the same conditions, there are varying degrees of drop in the removal percentage with higher initial concentrations of Pb, Cu, and Cd in the solution. Xiaoming Ma^90^ suggested that these observations could be attributed to the high collision efficiency between the metal ions and the adsorbent at lower initial concentrations. However, the adsorption capacities (q_e_) of the metal ions are no longer enhanced and remain constant or decrease after reaching saturation at higher concentrations (Table 4). The decrease in metal ion adsorption at higher concentrations can be attributed to the limited availability of vacant sites on the adsorption surfaces, thereby hindering further adsorption of the ions^91^.

From the foregoing discussion, it is concluded that the removal percentage of Pb, Cu, and Cd onto limestone and calcined limestone adsorbents depends on the initial concentrations and decreases with an increase in the initial concentration of metal ions. The difference in percentage removal of different heavy metal ions (Pb, Cu, and Cd) at the same experimental conditions may be attributed to the difference in the solubility products of their carbonates and hydroxides, chemical affinity, and ion exchange capacity with respect to the chemical functional group on the surface of the hybrid materials ^90^. Wierzba^92^ suggested that the main mechanism of heavy metal cations removal using limestone (CaCO_3_) and calcined limestone (CaO) is ion exchange, whereas the reaction with calcium carbonate results in the precipitation of poorly soluble carbonates and hydroxides of the examined heavy metals.

#### Co-removal of Pb, Cu, and Cd ions

Generally, heavy metals often coexist in the contaminated wastewater system and competitive behavior could affect the adsorption capacity of absorbents^93^. To evaluate the effect of competitive adsorption of coexisting Pb, Cu, and Cd on the adsorption capacity of limestone and calcined limestone, competitive adsorption tests of the ternary system were performed according to the following conditions:

1. Limestone adsorbent dose 0.8 g/L, initial concentrations 300 ppm for each heavy metal (Pb, Cu, and Cd), 30 min contact time, and pH 5.
2. calcined limestone adsorbent dose 0.16 g/L, initial concentration 600 ppm for each heavy metal ion (Pb, Cu, and Cd), contact time 30 min, and pH 5.

**Fig. 12:**
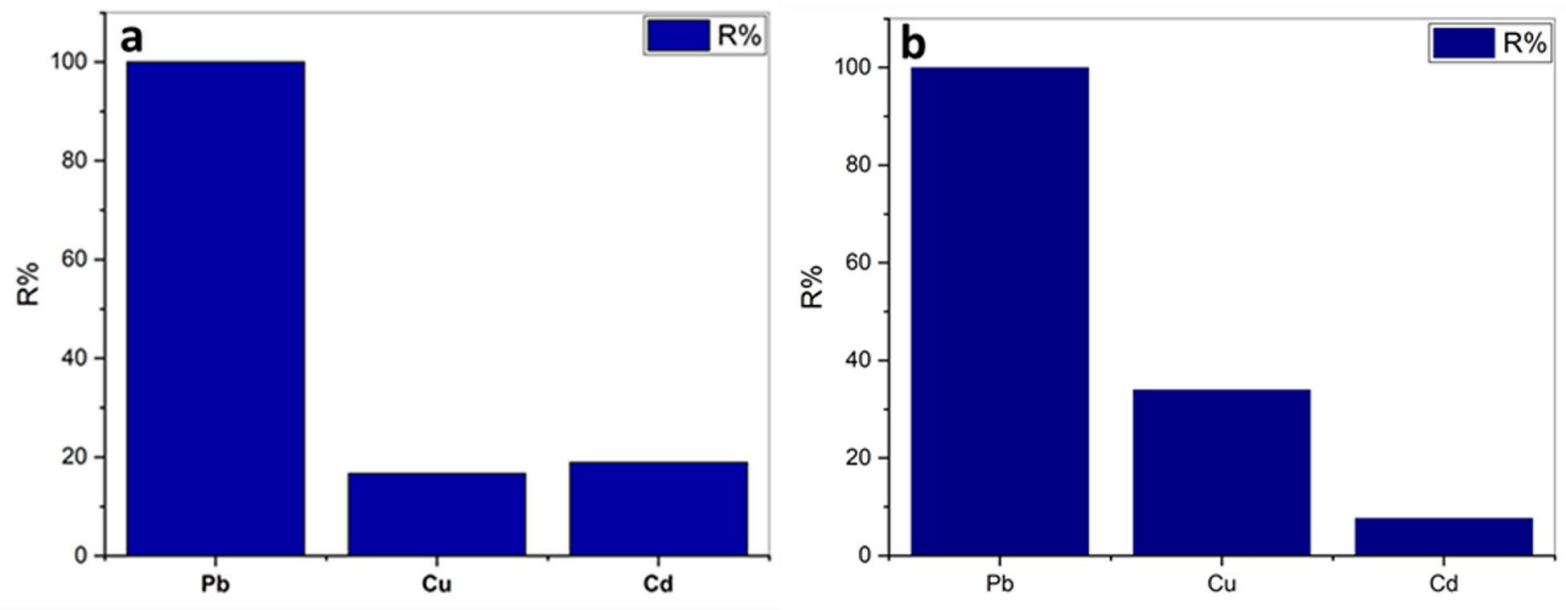
Co-sorption of Pb(II), Cu(II), and Cd(II) by (**a**) limestone (CaCO_3_) and (**b**) calcined (CaO) in ternary systems.

In ternary metals system of adsorption experiments, the adsorption capacities of Pb, Cu, and Cd metal ions were all lower than those in a single metal system. This difference in the adsorption capacities suggested that competitive adsorption occurred when Pb, Cu, and Cd metal ions coexisted in the solution. In Fig. 12a,b, it was observed that the affinity of limestone and calcined limestone towards Pb(II) was higher than Cu(II) and Cd(II). This is probably due to the fact that the solubility product of PbCO_3_ and Pb(OH)_2_ is smaller relative to Cu and Cd^92^. The affinity sequence of metal ions observed concerning limestone was Pb^2+^ > Cd^2+^ > Cu^2+^, while with respect to calcined limestone was Pb^2+^ > Cu^2+^ > Cd^2+^.

#### Isotherml models

Adsorption isotherms describe the equilibrium between the adsorbent and adsorbate in the adsorption process^94^. The interaction between the adsorbed molecules and the solid adsorbent is captured by several developed adsorption isotherm models. The Langmuir and Freundlich models, among others, are the most commonly employed isotherms in various adsorption processes^95,96^. The adsorption data obtained from the experiments in this study were subjected to analysis using both the Langmuir and Freundlich isotherm models.

The assumption made by the Langmuir isotherm model is that the surface of the adsorbent is uniform and consists of adsorption sites with the same energy level. This characteristic facilitates the adsorption of a monolayer through a chemisorption process^97^. Equation (4) represents the linear form of this model.

4 $$\frac{{C}_{e}}{{q}_{e}}=\frac{1}{b {q}_{max}}+\frac{{C}_{e}}{{q}_{max}}$$

To determine the values of q_max_ and b in the Langmuir isotherm model, a graph of (C_e_/q_e_) versus Ce can be utilized. Here, q_e_ represents the amount adsorbed in mg g^-1^, b denotes the equilibrium constant of adsorption, q_max_ signifies the maximum adsorption capacity in mg g^-1^, and C_e_ represents the equilibrium concentration in mg L^-1^. Equation (5) defines a dimensionless constant, known as the separation factor (R_L_)^98^.

5 $$\text{RL}=\frac{1}{(1+b {C}_{i})}$$

The separation factor (R_L_) is a useful parameter for characterizing the adsorption process. If R_L_ is greater than 1, the adsorption process is considered unfavorable. A value of R_L_ equal to 1 indicates a linear adsorption process. When R_L_ is between 0 and 1, the adsorption process is considered favorable. Finally, R_L_ equal to 0 signifies an irreversible adsorption process^98^.

The Freundlich isotherm model is employed to describe adsorption processes that occur on heterogeneous surfaces and active sites with varying energies, incorporating the concepts of multilayer adsorption and equilibrium^99^. Equation (6) represents the linear form of the Freundlich isotherm model^100,101^:

6 $$log qe=log Kf+\frac{1}{n} log Ce$$

By plotting log q_e_ against log C_e_, a linear relationship can be observed. From this plot, the Freundlich constants K_f_ and n can be determined from the intercept and slope, respectively. K_f_ represents the adsorption capacity, while n represents the adsorption intensity.

**Fig. 13:**
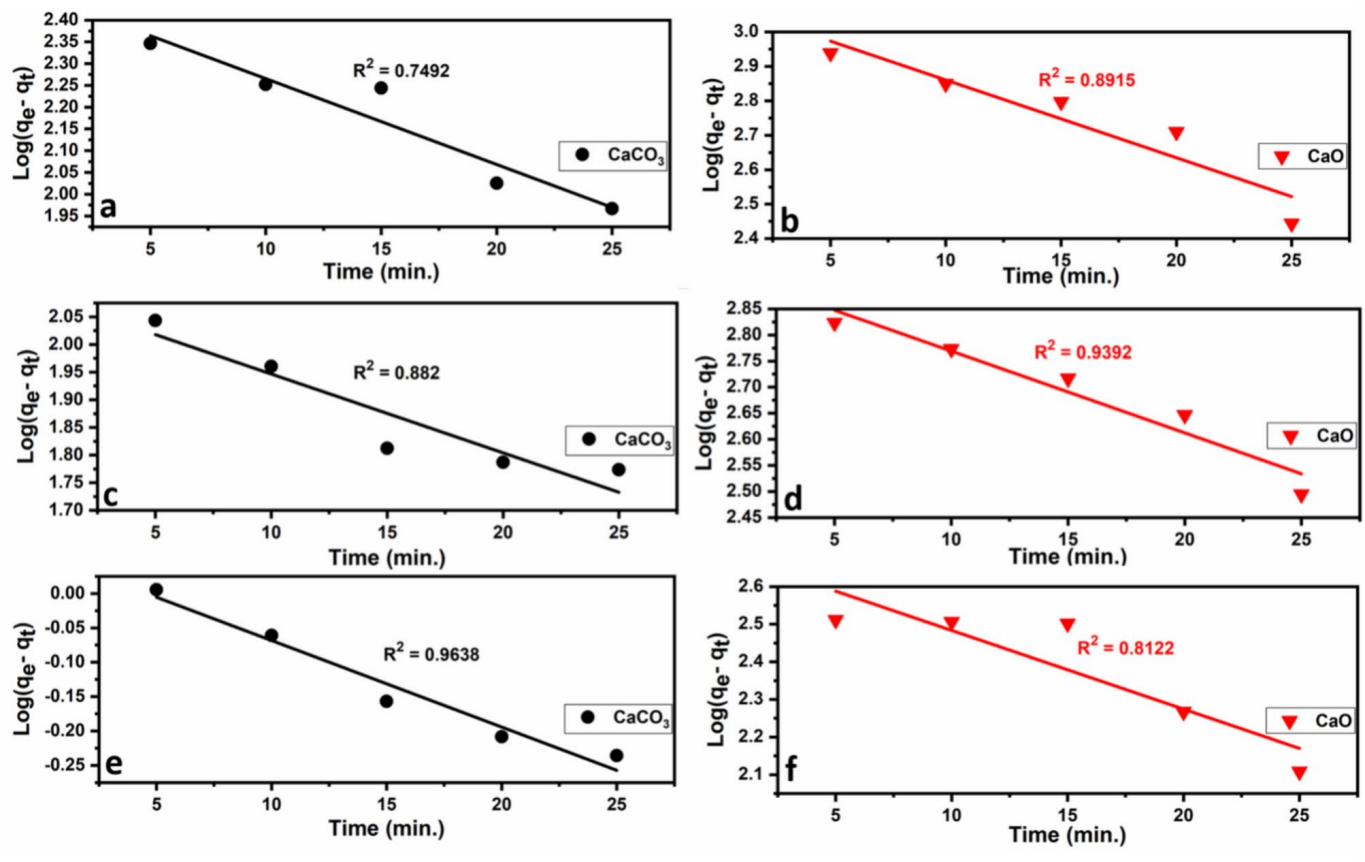
Langmuir isotherm plots for the adsorption of heavy metals (Pb^2+^ (**a**), Cu^2+^ (**b**, **c**), and Cd^2+^(**d**,** e**)) onto limestone (CaCO_3_) and calcined (CaO. According to the equation: $\frac{{C}_{e}}{{q}_{e}}=\frac{1}{b {q}_{max}}+\frac{{C}_{e}}{{q}_{max}}$^97^. P indicate the number of corresponding points.

**Fig. 14:**
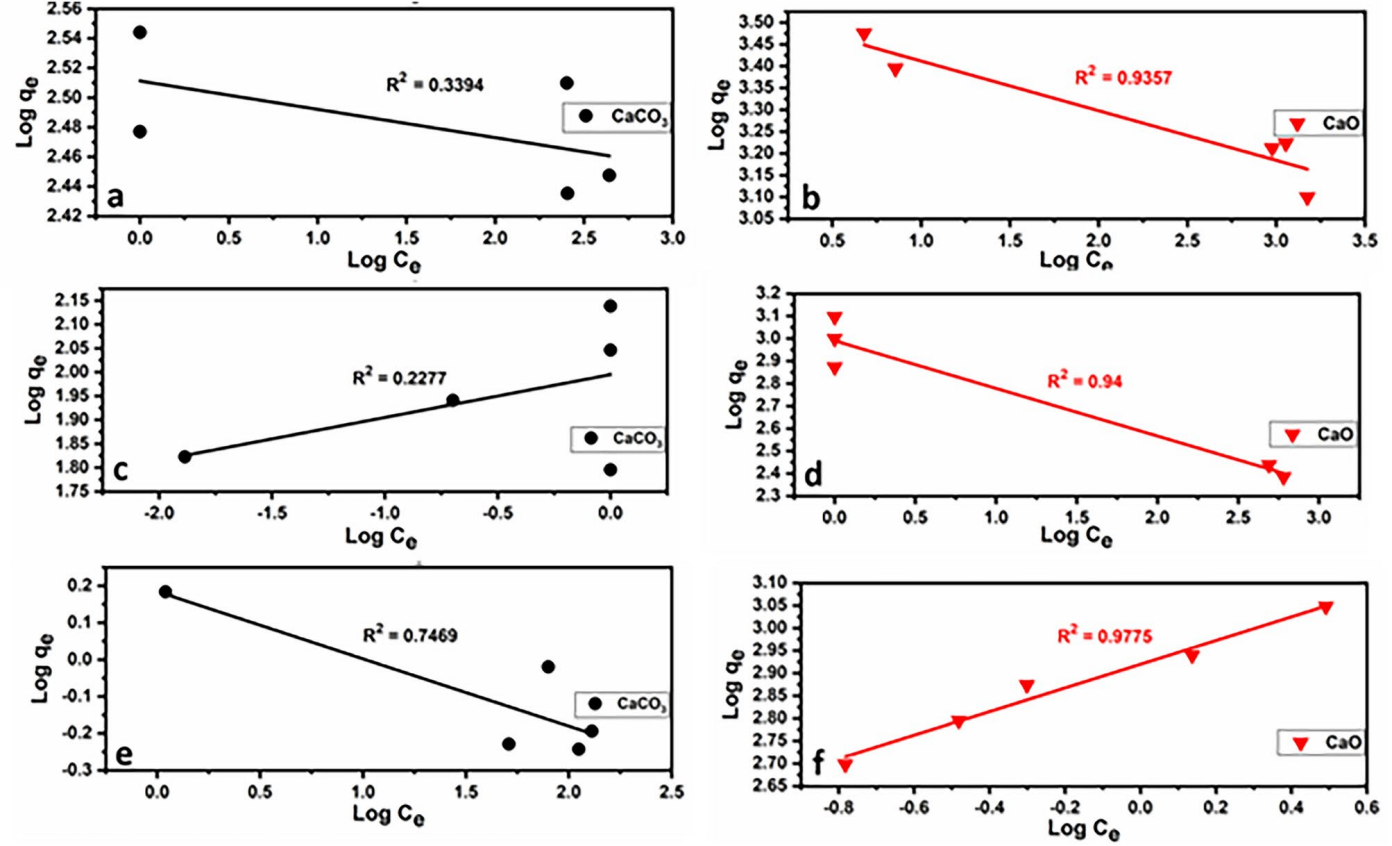
Freundlich isotherm plots for the adsorption of heavy metals (Pb^2+^ (**a**, **b**), Cu^2+^ (**c**, **d**), and Cd^2+^ (**e**, **f**)) onto limestone (CaCO_3_) and calcined limestone (CaO). According to the equation: log q_e_ = log K_f_ + $\frac{1}{\text{n}}$ log C_e_^101^.

As seen from Figs. 13 and 14 and Table 5, the heavy metal ions (Pb, Cu, and Cd) onto either limestone or calcined limestone fit well into the Langmuir adsorption model with correlation coefficients (R^2^) > 0.86, and 0 < R_L_ < 1 indicating favorable processes. This indicates physical monolayer adsorption. The fact that the Langmuir isotherm fits the experimental results very well may be due to the homogenous distribution of active sites on the adsorbent surfaces since the Langmuir isotherm equation assumes that the surface is homogenous^102^.

**Table 5:** Langmuir and Freundlich isotherm parameters and correlation coefficients for Pb^+2^, Cu^+2^, and Cd^+2^ adsorption onto CaCO_3_ and CaO.

Model	Parameters	CaCO_3_			CaO		
		Pb^+2^	Cu^+2^	Cd^+2^	Pb^+2^	Cu^+2^	Cd^+2^
Langmuir	q_max_ (mg/g)	270.3	73.53	0.61	1666.6	769.2	1250
	K_L_ (b)	0.086	6.18	0.5	1.5	13	4
	R_L_	0.019–0.011	0.003–0.001	0.038**–**0.013	0.0007–0.0003	0.0007–0.00011	0.001–0.0005
	R^2^	0.957	0.9845	0.9881	0.9997	0.862	0.999
Freundlich	K_f_	324.56	98.22	1.53	3409.57	978.1	832.72
	n	− 52.35	14.92	− 5.49	− 8.1	− 4.73	3.8
	R^2^	0.3394	0.2277	0.7469	0.9357	0.94	0.9775

#### Kinetic models

The rate of adsorption of metal ions on the adsorbents is determined by kinetics, which helps to establish the minimum contact time required for the adsorbent to achieve suitable efficiency. In this study, the adsorption mechanisms of Pb^2+^, Cu^2+^, and Cd^2+^ onto limestone and calcined limestone were explained using the pseudo-first order, pseudo-second order, and intra-particle diffusion kinetic model^103–105^. Below are the linear equations corresponding to each kinetic model:

The pseudo-first-order kinetics is given by Eq. (7):

7 $$\text{log}\left({q}_{e}-{q}_{t}\right)={\text{log}q}_{e}-\frac{({k}_{1}t)}{2.303}$$

In the pseudo-first-order adsorption model, the rate constant is represented by k_1_ (mg/g min), while q_e_ (mg/g) and q_t_ (mg/g) refer to the amounts of heavy metal anions that are adsorbed at equilibrium and at a given time t, respectively.

To apply the pseudo-second-order equation, Eq. (8) is utilized in the given form:

8 $$\frac{t}{{q}_{t}}= \frac{1}{({k}_{2 }{q}_{e}^{2})}+\frac{t}{{q}_{e}}$$

The second-order rate constant, k_2_ (mg/g min), is determined from the plot of t/q_t_ versus t, as illustrated in Fig. 16. Furthermore, Eq. (9) can be employed to calculate the initial adsorption rate h (mg/g min)^104^.

9 $$h = k_{2} \cdot q_{e}^{2}$$

**Fig. 15:**
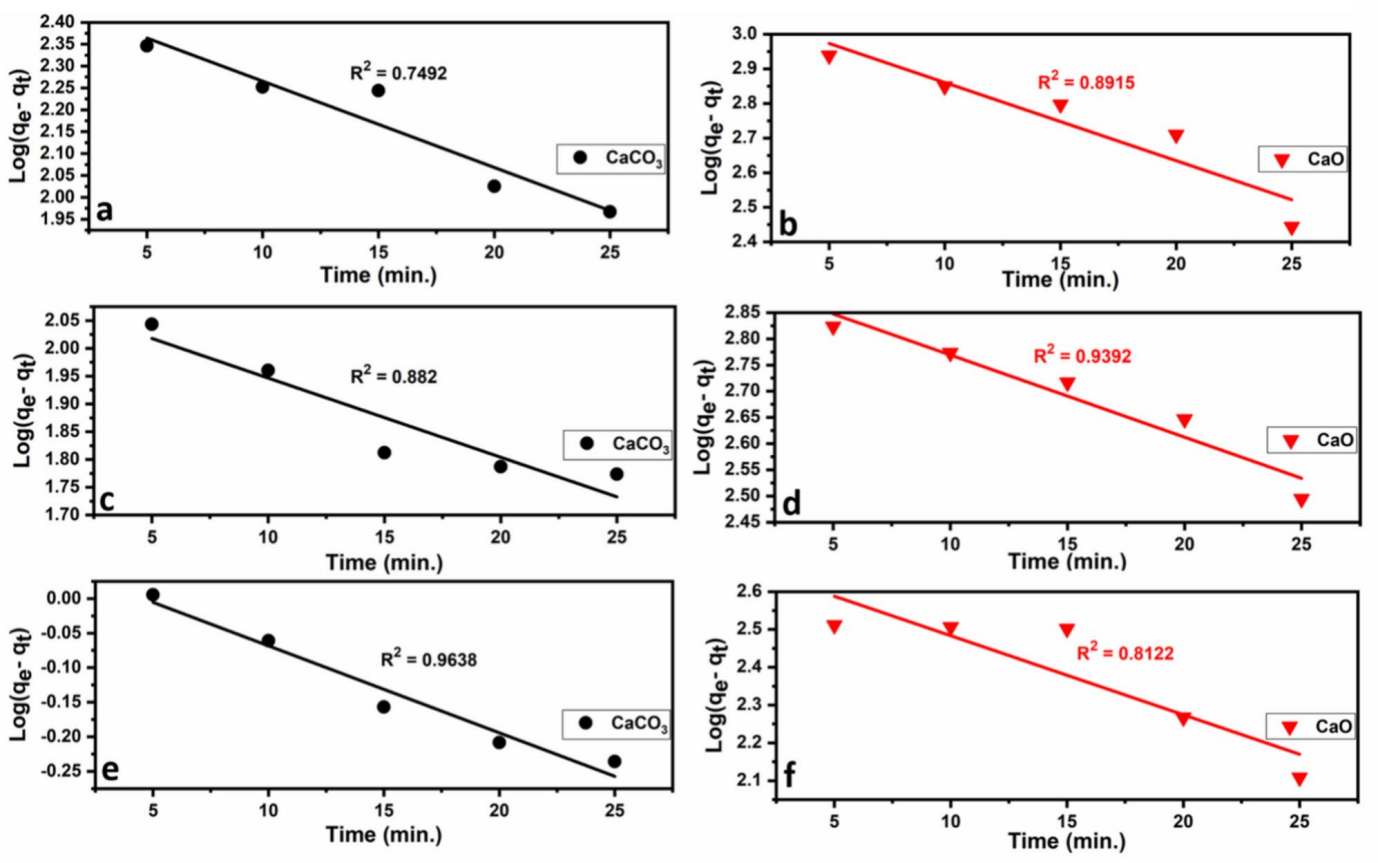
Pseudo-first-order kinetic plots for the heavy metal ions (Pb^2+^ (**a**), Cu^2+^ (**b**), Cd^2+^ (**c**, **d**)) onto limestone (CaCO_3_) and calcined limestone (CaO). According to the equation: $\text{log}$ (${\text{q}}_{\text{e}}$ − ${\text{q}}_{\text{t}}$) = ${\text{logq}}_{\text{e}}- \frac{({\text{k}}_{1}\text{t})}{2.303}$^103^.

**Fig. 16:**
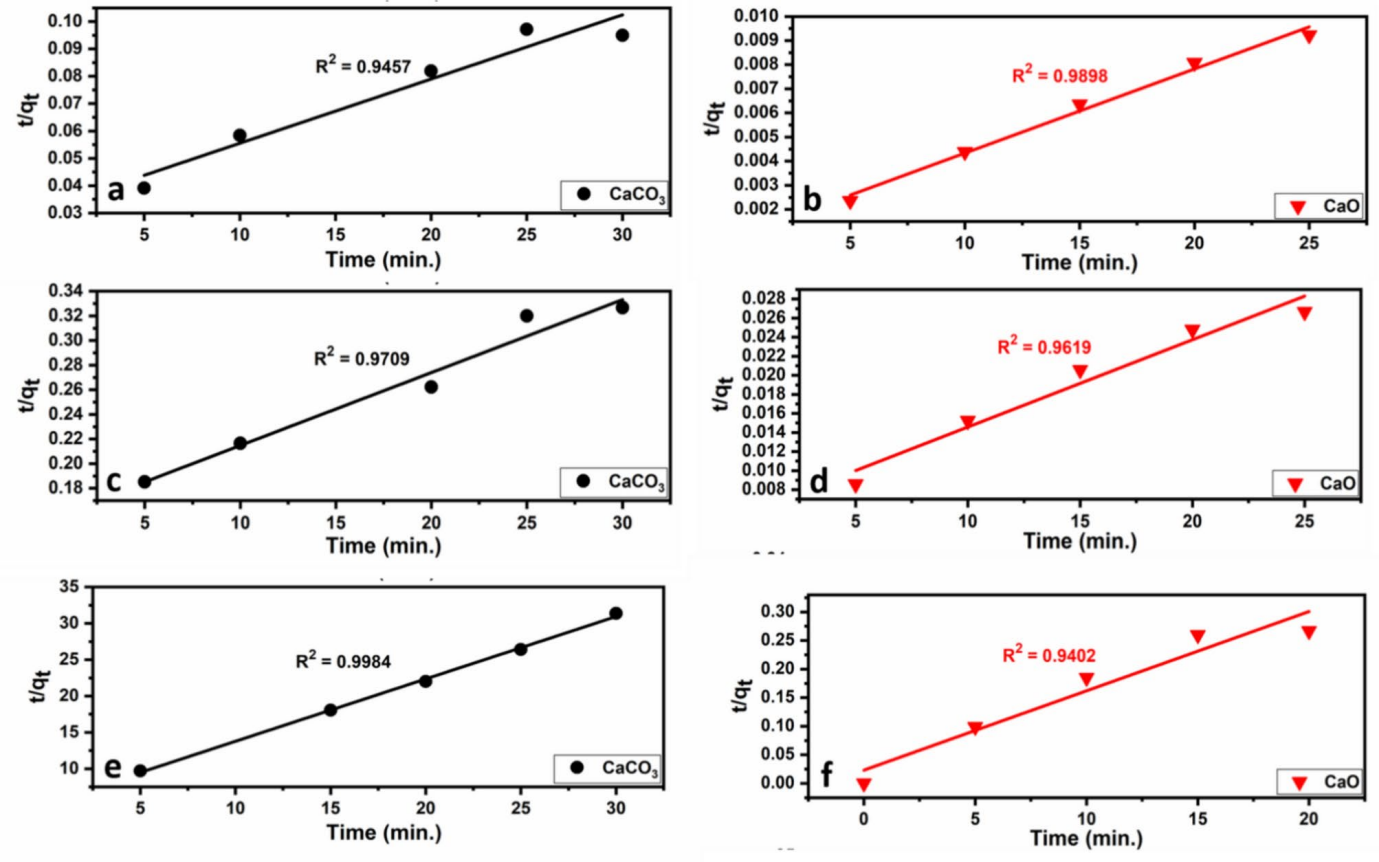
Pseudo-second-order kinetic plots for the heavy metal ions (Pb^2+^ (**a**, **b**), Cu^2+^ (**c**, **d**), and Cd^2+^ (**e**, **f**)) on to limestone (CaCO_3_) and calcined limestone (CaO). According to the equation: $\frac{\text{t}}{{\text{q}}_{\text{t}}}= \frac{1}{({\text{k}}_{2 }{\text{q}}_{\text{e}}^{2})}+\frac{\text{t}}{{\text{q}}_{\text{e}}}$^104^.

The results of applying the kinetic models are given in Figs. 15 and 16 and Table 6. The pseudo-second-order model fits quite well with the experimental data of Pb^2+^, Cu^2+^, and Cd^2+^ adsorption onto limestone and calcined limestone. This model confirms the chemisorption of Pb(II), Cu(II), and Cd(II) onto both limestone and calcined limestone^106^.

**Table 6:** Parameters of the studied Pseudo-First-order and Pseudo-Second-order kinetic models of CaCO_3_ and CaO adsorbents.

Model	Parameters	CaCO_3_			CaO		
		Pb^+2^	Cu^+2^	Cd^+2^	Pb^+2^	Cu^+2^	Cd^+2^
Pseudo-first-order	K_1_ (mg/g min)	0.089	0.033	0.029	0.052	0.036	0.048
	q_e_ (mg/g)	484.06	122.8	1.14	1220.4	843.53	492.5
	R^2^	0.7492	0.882	0.9638	0.8915	0.9392	0.8122
Pseudo-second-order	K_2_ (mg/g min)	0.00016	0.00022	0.14	0.0001	0.00015	0.0083
	q_e_ (mg/g)	434.78	169.49	1.165	3333.3	1111.1	71.9
	h (mg/g min)	31.2	6.43	0.19	1111.1	185.19	43.1
	R^2^	0.9457	0.9709	0.9984	0.9898	0.9619	0.9402

##### Intra-particle diffusion model

IP model has been widely applied to examine the rate-limiting step during adsorption. Weber^107^ observed that in most adsorption processes the uptake of adsorbates varies proportionally with the square root of time (t^1/2^)^108^.

The intra-particle diffusion model is expressed by the following Eq. (10)^109^:

10 $$q_{t} = k_{IPD} t^{1/2} + C$$

K_IPD_ = Intra-particle diffusion constant (mg/min^1/2^. g), C = Thickness of boundary layer (intercept).

**Fig. 17:**
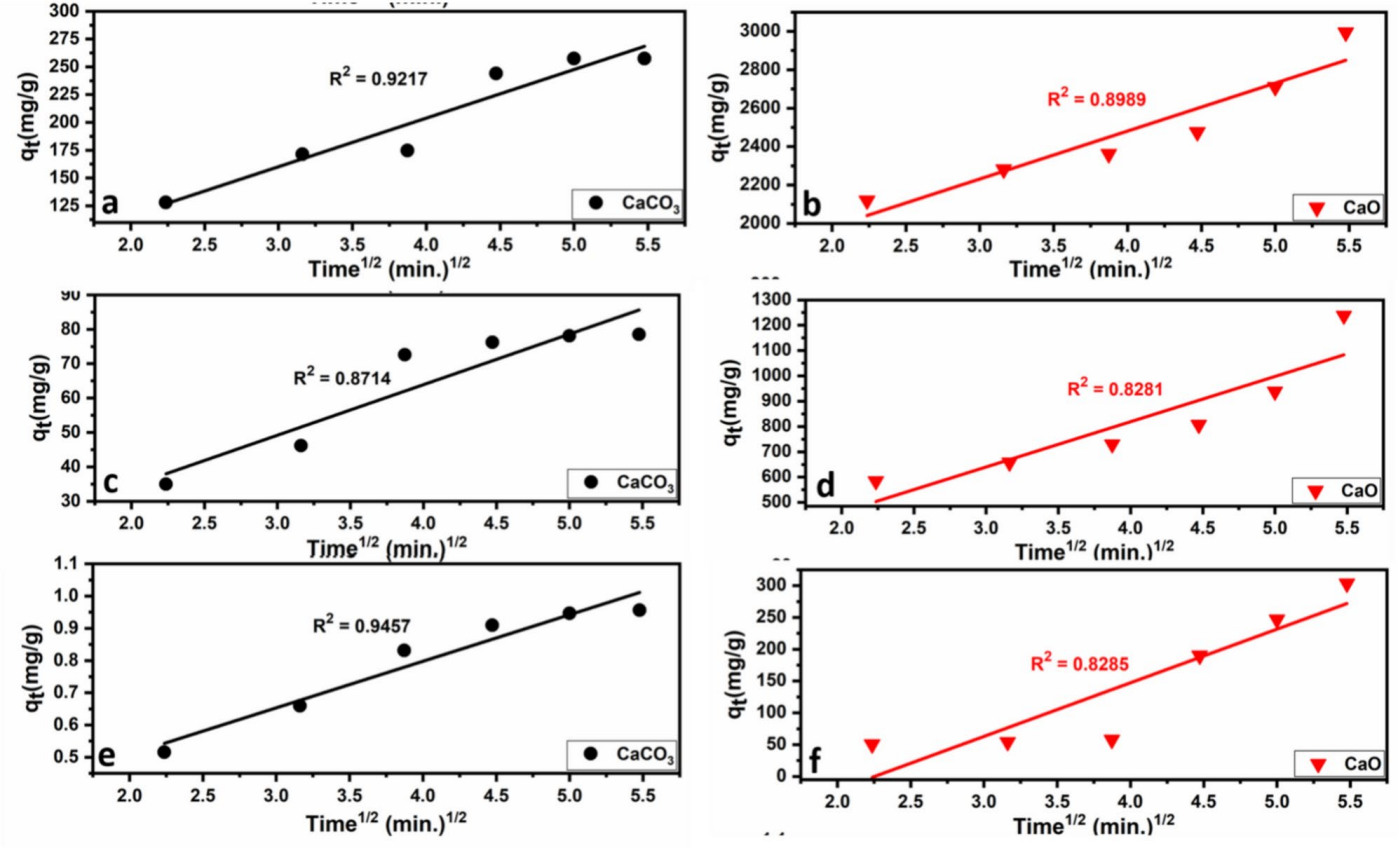
Intra-particle diffusion models for Pb^2+^ (**a**, **b**), Cu^2+^ (**c**, **d**), and Cd^2+^ (**e**, **f**) onto limestone (CaCO_3_) and calcined limestone (CaO). According to the equation: q_t_ = k_IPD_ t^1/2^ + C ^109^.

The results of the intra-particle diffusion model are provided in Table 7 and Fig. 17.

**Table 7:** Parameters of the studied Intra-particle diffusion models of CaCO_3_ and CaO adsorbents.

Model	Parameters	CaCO_3_			CaO		
		Pb^+2^	Cu^+2^	Cd^+2^	Pb^+2^	Cu^+2^	Cd^+2^
Intra-particle diffusion	K_IPD_ (mg/min^1/2^.g)	43.717	14.699	0.1445	249.27	178.74	84.3
	C	28.99	5.1088	0.2199	1484	103.96	-190
	R^2^	0.9217	0.8714	0.9457	0.8989	0.8281	0.8285

The summary of kinetic model constant values for Pb, Cu, and Cd adsorption onto limestone and calcined limestone showed that the pseudo-second-order kinetic model fit well with the experimental data compared to pseudo-first-order and intra-particle diffusion models.

## Conclusion

The present study supports the adsorption of heavy metals Pb, Cu, and Cd using limestone wastes and CaO derived from the calcination of limestone. The mechanism of heavy metals adsorption is ion exchange, whereas the reactions of heavy metals with limestone and calcined limestone result in the precipitation of poorly soluble carbonate and hydroxides. The Langmuir isotherm was preferred to describe the adsorption characteristics of Pb, Cu, and Cd ions onto limestone (CaCO_3_) and calcined limestone (CaO). The model suggested that the adsorption processes were a single molecule layer adsorption. The application of kinetic models to the experimental results of the heavy metals adsorption onto limestone (CaCO_3_) and calcined limestone (CaO) showed that the pseudo-second-order model fit well with the experimental data. This model confirms the chemisorption of Pb, Cu, and Cd onto limestone wastes (CaCO_3_) and calcined limestone (CaO). In terms of metal removal affinity, the order of preference for calcined limestone was Pb > Cu > Cd, while for limestone was Pb > Cd > Cu.

## Data Availability

The data and chemical analysis that present in the research article are available with the corresponding author (Moneim) in reasonable request.

## References

[1] Barhoumi, S., Messaoudi, I., Deli, T., Saïd, K. & Kerkeni, A. Cadmium bioaccumulation in three benthic fish species, Salaria basilisca, Zosterisessor ophiocephalus and Solea vulgaris collected from the Gulf of Gabes in Tunisia. *J. Environ. Sci.* **21**(7), 980–984 (2009). 10.1016/s1001-0742(08)62371-2 19862966

[2] Eloussaief, M. & Benzina, M. Efficiency of natural and acid-activated clays in the removal of Pb(II) from aqueous solutions. *J. Hazard Mater.* **178**(1–3), 753–757 (2010). 20189300 10.1016/j.jhazmat.2010.02.004

[3] Gardea-Torresdey, J. L., Peralta-Videa, J. R., De La Rosa, G. & Parsons, J. G. Phytoremediation of heavy metals and study of the metal coordination by X-ray absorption spectroscopy. *Coord. Chem. Rev.* **249**(17-18 SPEC. ISS.), 1797–1810 (2005).

[4] Hussein, H., Farag, S., Kandil, K. & Moawad, H. Tolerance and uptake of heavy metals by Pseudomonads. *Process Biochem.* **40**(2), 955–961 (2005).

[5] Martín-González, A., Díaz, S., Borniquel, S., Gallego, A. & Gutiérrez, J. C. Cytotoxicity and bioaccumulation of heavy metals by ciliated protozoa isolated from urban wastewater treatment plants. *Res. Microbiol.* **157**(2), 108–118 (2006). 16129584 10.1016/j.resmic.2005.06.005

[6] Sdiri, A., Higashi, T., Jamoussi, F. & Bouaziz, S. Effects of impurities on the removal of heavy metals by natural limestones in aqueous systems. *J. Environ. Manage.* **93**(1), 245–253 (2012). 22054591 10.1016/j.jenvman.2011.08.002

[7] Adriano, D. C., Wenzel, W. W., Vangronsveld, J. & Bolan, N. S. Role of assisted natural remediation in environmental cleanup. *Geoderma.* **122**(2-4 SPEC. IIS.), 121–142 (2004).

[8] Qin, F. et al. Mechanisms of competitive adsorption of Pb, Cu, and Cd on peat. *Environ. Pollut.* **144**(2), 669–680 (2006). 16616404 10.1016/j.envpol.2005.12.036

[9] Ahmad, K., Bhatti, I.A., Muneer, M., Iqbal, M. & Iqbal, Z. Removal of heavy metals (Zn, Cr, Pb, Cd, Cu and Fe) in aqueous media by calcium carbonate as an adsorbent. **2**, 48–53 (2012).

[10] Rashed, M. N., Mohamed, A. R. & Awadallah, M. A. Chemically activated phosphate slime as adsorbent for heavy metals removal from polluted water. *Int. J. Environ. Waste Manag.* **16**(2), 145–165 (2015).

[11] Okoye, A. I., Ejikeme, P. M. & Onukwuli, O. D. Lead removal from wastewater using fluted pumpkin seed shell activated carbon: Adsorption modeling and kinetics. *Int. J. Environ. Sci. Technol.* **7**(4), 793–800 (2010).

[12] Boparai, H. K., Joseph, M. & O’Carroll, D. M. Kinetics and thermodynamics of cadmium ion removal by adsorption onto nano zerovalent iron particles. *J. Hazard Mater.* **186**(1), 458–465 (2011). 21130566 10.1016/j.jhazmat.2010.11.029

[13] Srivastava, V. C., Mall, I. D. & Mishra, I. M. Equilibrium modelling of single and binary adsorption of cadmium and nickel onto bagasse fly ash. *Chem. Eng. J.* **117**(1), 79–91 (2006).

[14] Fu, F. & Wang, Q. Removal of heavy metal ions from wastewaters: A review. *J. Environ. Manage.* **92**(3), 407–418 (2011). 21138785 10.1016/j.jenvman.2010.11.011

[15] Ugochukwu, U. C. et al. Heavy metal contamination of soil, sediment and water due to galena mining in Ebonyi State Nigeria: Economic costs of pollution based on exposure health risks. *J. Environ. Manage.* **321**, 115864 (2022). 35981502 10.1016/j.jenvman.2022.115864

[16] Chen, Q., Yao, Y., Li, X., Lu, J., Zhou, J. & Huang, Z. Comparison of heavy metal removals from aqueous solutions by chemical precipitation and characteristics of precipitates. *J. Water Process. Eng*. 289–300 (2018).

[17] Song, S. et al. Arsenic removal from high-arsenic water by enhanced coagulation with ferric ions and coarse calcite. *Water Res.* **40**(2), 364–372 (2006). 16352327 10.1016/j.watres.2005.09.046

[18] Brbooti, M.M., Abid, Balasim, A. & Al-shuwaiki, N.M. Removal of heavy metals using chemicals precipitation. *Eng. Technol. J*. **29**(3) (2011).

[19] Matlock, M. M., Howerton, B. S. & Atwood, D. A. Chemical precipitation of heavy metals from acid mine drainage. *Water Res.* **36**(19), 4757–4764 (2002). 12448518 10.1016/s0043-1354(02)00149-5

[20] Al-Rashdi, B. A. M., Johnson, D. J. & Hilal, N. Removal of heavy metal ions by nanofiltration. *Desalination.* **315**, 2–17 (2013).

[21] Blöcher, C. et al. Hybrid flotation—Membrane filtration process for the removal of heavy metal ions from wastewater. *Water Res.* **37**(16), 4018–4026 (2003). 12909122 10.1016/S0043-1354(03)00314-2

[22] Tavakoli, O., Goodarzi, V. & Reza, M. Competitive removal of heavy metal ions from squid oil under isothermal condition by CR11 chelate ion exchanger. *J. Hazard Mater.* **334**, 256–266 (2017). 28419932 10.1016/j.jhazmat.2017.04.023

[23] Wang, Z., Feng, Y., Hao, X., Huang, W. & Feng, X. A novel potential-responsive ion exchange film system for heavy metal removal. *J. Mater. Chem. A.* **2**(26), 10263–10272 (2014).

[24] Shi, M. et al. Construction and evaluation of a novel three-electrode capacitive deionization system with high desalination performance. *Sep. Purif. Technol.* **273**, 118976 (2021).

[25] Gürses, A., Yalçin, M. & Dogar, C. Removal of Remazol red RB by using AI (III) as coagulant-flocculant: Effect of some variables on settling velocity. *Water Air Soil. Pollut.* **146**(1–4), 297–318 (2003).

[26] Celis, R., Hermosín, C. M. & Cornejo, J. Heavy metal adsorption by functionalized clays. *Environ. Sci. Technol.* **34**(21), 4593–4599 (2000).

[27] Dardir, F.M., Ahmed, E.A., Soliman, M.F. & Abukhadra, M.R. Green synthesis of phillipsite from natural microcline for use as an adsorbent for Cu (II), Cd (II), Pb (II), and methylene blue dye from polluted water. *Euro-Mediterranean J. Environ. Integr*. 1–10 (2024).

[28] Zhang, R., Richardson, J.J., Masters, A.F., Yun, G., Liang, K. & Maschmeyer T. Effective removal of toxic heavy metal ions from aqueous solution by CaCO3 microparticles. *Water Air Soil Pollut*. **229**(4) (2018).

[29] Baskaralingam, P., Pulikesi, M., Elango, D., Ramamurthi, V. & Sivanesan, S. Adsorption of acid dye onto organobentonite. *J. Hazard Mater.* **128**(2–3), 138–144 (2006). 16360263 10.1016/j.jhazmat.2005.07.049

[30] Qasem, N. A. A., Mohammed, R. H. & Lawal, D. U. Removal of heavy metal ions from wastewater: A comprehensive and critical review. *NPJ Clean Water.* **4**(1), 1–15 (2021).

[31] Amuda, O. S., Giwa, A. A. & Bello, I. A. Removal of heavy metal from industrial wastewater using modified activated coconut shell carbon. *Biochem. Eng. J.* **36**(2), 174–181 (2007).

[32] Mandal, S., Calderon, J., Marpu, S. B., Omary, M. A. & Shi, S. Q. Mesoporous activated carbon as a green adsorbent for the removal of heavy metals and Congo red: Characterization, adsorption kinetics, and isotherm studies. *J. Contam. Hydrol.* **243**, 103869 (2021). 34418820 10.1016/j.jconhyd.2021.103869

[33] Kobya, M., Demirbas, E., Senturk, E. & Ince, M. Adsorption of heavy metal ions from aqueous solutions by activated carbon prepared from apricot stone. *Bioresour. Technol.* **96**(13), 1518–1521 (2005). 15939281 10.1016/j.biortech.2004.12.005

[34] Stafiej, A. & Pyrzynska, K. Adsorption of heavy metal ions with carbon nanotubes. *Sep. Purif. Technol.* **58**(1), 49–52 (2007).

[35] Ricco, R. et al. Lead(II) uptake by aluminium based magnetic framework composites (MFCs) in water. *J. Mater. Chem. A.* **3**(39), 19822–19831 (2015).

[36] Dardir, F. M., Ahmed, E. A., Soliman, M. F. & Abukhadra, M. R. Removal of Pb+2 and Cd+2 from aqueous solution by using faujasite. *Int. J. Miner. Process Extr. Metall.* **8**(1), 1–8 (2023).

[37] Gupta, V. K., Agarwal, S. & Saleh, T. A. Synthesis and characterization of alumina-coated carbon nanotubes and their application for lead removal. *J. Hazard Mater.* **185**(1), 17–23 (2011). 20888691 10.1016/j.jhazmat.2010.08.053

[38] Babel, S. & Kurniawan, T. A. Low-cost adsorbents for heavy metals uptake from contaminated water: A review. *J. Hazard Mater.* **97**(1–3), 219–243 (2003). 12573840 10.1016/s0304-3894(02)00263-7

[39] Topare, N. S. & Wadgaonkar, V. S. A review on application of low-cost adsorbents for heavy metals removal from wastewater. *Mater. Today Proc.* **77**, 8–18 (2022).

[40] Thakur, S. K., Tomar, N. K. & Pandeya, S. B. Influence of phosphate on cadmium sorption by calcium carbonate. *Geoderma.* **130**(3–4), 240–249 (2006).

[41] Larous, S., Meniai, A. H. & Lehocine, B. M. Experimental study of the removal of copper from aqueous solutions by adsorption using sawdust. *Desalination.* **185**(1–3), 483–490 (2005).

[42] Feng, D., Van Deventer, J. S. J. & Aldrich, C. Removal of pollutants from acid mine wastewater using metallurgical by-product slags. *Sep. Purif. Technol.* **40**(1), 61–67 (2004).

[43] Liu, C. & Huang, P. M. Kinetics of lead adsorption by iron oxides formed under the influence of citrate. *Geochim. Cosmochim. Acta.* **67**(5), 1045–1054 (2003).

[44] Erdem, M. & Özverdi, A. Lead adsorption from aqueous solution onto siderite. *Sep. Purif. Technol.* **42**(3), 259–264 (2005).

[45] Wang, Y. & Reardon, E. J. A siderite/limestone reactor to remove arsenic and cadmium from wastewaters. *Appl. Geochem.* **16**(9–10), 1241–1249 (2001).

[46] Elouear, Z. et al. Heavy metal removal from aqueous solutions by activated phosphate rock. *J. Hazard. Mater.* **156**(1–3), 412–420 (2008). 18242833 10.1016/j.jhazmat.2007.12.036

[47] Mouflih, M., Aklil, A. & Sebti, S. Removal of lead from aqueous solutions by activated phosphate. *J. Hazard Mater.* **119**(1–3), 183–188 (2005). 15752864 10.1016/j.jhazmat.2004.12.005

[48] Perić, J., Trgo, M. & Medvidović, V. N. Removal of zinc, copper and lead by natural zeolite—A comparison of adsorption isotherms. *Water Res.* **38**(7), 1893–1899 (2004). 15026244 10.1016/j.watres.2003.12.035

[49] Park, J. H. et al. Characteristics of adsorption behavior of potentially toxic metals by biochar derived from fallen leaves (Platanus) and its mechanism. *Sustain. Chem. Pharm.* **29**, 100776 (2022).

[50] Hu, X., Zhang, R., Xia, B., et al. Effect of pyrolysis temperature on removal efficiency and mechanisms of Hg(II), Cd(II), and Pb (II) by maize straw biochar. *Sustain*. **14**(15) (2022).

[51] Wang, H., Zhang, M. & Lv, Q. Influence of pyrolysis temperature on cadmium removal capacity and mechanism by maize straw and platanus leaves biochars. *Int. J. Environ. Res. Public Health.* **16**(5), 845 (2019). 30857159 10.3390/ijerph16050845 PMC6427420

[52] Valverde, J. M. & Medina, S. Crystallographic transformation of limestone during calcination under CO2. *Phys. Chem. Chem. Phys.* **17**(34), 21912–21926 (2015). 26235797 10.1039/c5cp02715b

[53] Rodriguez-Navarro, C., Ruiz-Agudo, E., Luque, A., Rodriguez-Navarro, A. B. & Ortega-Huertas, M. Thermal decomposition of calcite: Mechanisms of formation and textural evolution of CaO nanocrystals. *Am. Mineral.* **94**(4), 578–593 (2009).

[54] Kim, M. S., Jun, Y., Lee, C. & Oh, J. E. Use of CaO as an activator for producing a price-competitive non-cement structural binder using ground granulated blast furnace slag. *Cem. Concr. Res.* **54**, 208–214 (2013).

[55] Merrikhpour, H. & Jalali, M. Waste calcite sludge as an adsorbent for the removal of cadmium, copper, lead, and zinc from aqueous solutions. *Clean Technol. Environ. Policy.* **14**(5), 845–855 (2012).

[56] Chang, M. C. & Tai, C. Y. Effect of the magnetic field on the growth rate of aragonite and the precipitation of CaCO3. *Chem. Eng. J.* **164**(1), 1–9 (2010).

[57] Zhang, Z., Xie, Y., Xu, X., Pan, H. & Tang, R. Transformation of amorphous calcium carbonate into aragonite. *J. Cryst. Growth.* **343**(1), 62–67 (2012).

[58] Kamalanathan, P. et al. Synthesis and sintering of hydroxyapatite derived from eggshells as a calcium precursor. *Ceram. Int.* **40**(PB), 16349–16359 (2014).

[59] Gunasekaran, S., Anbalagan, G. & Pandi, S. Raman and infrared spectra of carbonates of calcite structure. *J. Raman Spectrosc.* **37**(9), 892–899 (2006).

[60] Liu, R., Guan, Y., Chen, L. & Lian, B. Adsorption and desorption characteristics of Cd2+ and Pb2+ by micro and nano-sized biogenic CaCO3. *Front. Microbiol.* **9**, 41 (2018). 29434577 10.3389/fmicb.2018.00041 PMC5790784

[61] Farcas, F. & Touzé, P. Infrared Fourier Transform (IRFT) spectrometry. A valuable technique for characterizing cement. *Bull des Lab des Ponts Chaussées.* **230**(4350), 77–88 (2001).

[62] Mirghiasi, Z., Bakhtiari, F., Darezereshki, E. & Esmaeilzadeh, E. Preparation and characterization of CaO nanoparticles from Ca(OH)2 by direct thermal decomposition method. *J. Ind. Eng. Chem.* **20**(1), 113–117 (2014).

[63] He, Y. et al. Separatable MoS2 loaded biochar/CaCO3/Alginate gel beads for selective and efficient removal of Pb(II) from aqueous solution. *Sep. Purif. Technol.* **303**, 122212 (2022).

[64] Wang, S. et al. Highly adsorptive pristine and magnetic biochars prepared from crayfish shell for removal of Cu(II) and Pb(II). *J. Taiwan Inst. Chem. Eng.* **127**, 175–185 (2021).

[65] Ge, S. et al. Exploring adsorption capacity and mechanisms involved in cadmium removal from aqueous solutions by biochar derived from euhalophyte. *Sci. Rep.* **14**(1), 1–11 (2024). 38172293 10.1038/s41598-023-50525-2 PMC10764732

[66] Kasirajan, R., Bekele, A. & Girma, E. Adsorption of lead (Pb-II) using CaO-NPs synthesized by solgel process from hen eggshell: Response surface methodology for modeling, optimization and kinetic studies. *S. Afr. J. Chem. Eng.* **40**(1), 209–229 (2022).

[67] Chinthakuntla, A., Thatikayala, D., Rao, K.V., Chakra, C.S. & Kumar, M.K. Calcium oxide nano particles synthesized from chicken egg shells by physical method. *Int. Conf. Emerg. Technol. Mech. Sci.*72–75 (2014).

[68] Dümichen, E. et al. Analysis of polyethylene microplastics in environmental samples, using a thermal decomposition method. *Water Res.* **85**, 451–457 (2015). 26376022 10.1016/j.watres.2015.09.002

[69] Luo, X., Song, X., Cao, Y., Song, L. & Bu, X. Investigation of calcium carbonate synthesized by steamed ammonia liquid waste without use of additives. *RSC Adv.* **10**(13), 7976–7986 (2020). 35492173 10.1039/c9ra10460g PMC9049945

[70] Wang, H., Zhai, L., Li, Y. & Shi, T. Preparation of irregular mesoporous hydroxyapatite. *Mater. Res. Bull.* **43**(6), 1607–1614 (2008).

[71] Zhong, Q. & Bjerle, I. Calcination kinetics of limestone and the microstructure of nascent CaO. *Thermochim Acta.* **223**(C), 109–120 (1993).

[72] Bourli, N., Kokkaliari, M., Iliopoulos, I. & Zelilidis, A. Approaching the porosity values of Cretaceous carbonate deposits in Araxos peninsula and Kastos Island (North-Western Peloponnese, Greece) for the characterization of reservoir quality. *Bull. Geol. Soc. Greece Spec. Publ*. 7 (2019).

[73] Zhao, Z. et al. Surfactant-assisted solvo- or hydrothermal fabrication and characterization of high-surface-area porous calcium carbonate with multiple morphologies. *Microporous Mesoporous Mater.* **138**(1–3), 191–199 (2011).

[74] Paredes-Laverde, M. et al. Removal of norfloxacin in deionized, municipal water and urine using rice (Oryza sativa) and coffee (Coffea arabica) husk wastes as natural adsorbents. *J. Environ. Manag.* **213**, 98–108 (2018). 10.1016/j.jenvman.2018.02.047 29482094

[75] García-Sánchez, A. & Álvarez-Ayuso, E. Sorption of Zn, Cd and Cr on calcite. Application to purification of industrial wastewaters. *Miner. Eng.* **15**(7), 539–547 (2002).

[76] Malkoc, E. Ni(II) removal from aqueous solutions using cone biomass of Thuja orientalis. *J. Hazard Mater.* **137**(2), 899–908 (2006). 16621254 10.1016/j.jhazmat.2006.03.004

[77] Ertugay, N. & Malkoc, E. Adsorption isotherm, kinetic, and thermodynamic studies for methylene blue from aqueous solution by needles of pinus sylvestris L. *Polish J. Environ. Stud.* **23**(6), 1995–2006 (2014).

[78] Hu, Z., Chen, H., Ji, F. & Yuan, S. Removal of Congo Red from aqueous solution by cattail root. *J. Hazard Mater.* **173**(1–3), 292–297 (2010). 19765895 10.1016/j.jhazmat.2009.08.082

[79] Kumar, P. Y., King, P. & Prasad, V. S. R. K. Equilibrium and kinetic studies for the biosorption system of copper(II) ion from aqueous solution using Tectona grandis L.f. leaves powder. *J. Hazard Mater.* **137**(2), 1211–1217 (2006). 16704905 10.1016/j.jhazmat.2006.04.006

[80] Bayramoǧlu, G. & Arica, Y. M. Construction a hybrid biosorbent using Scenedesmus quadricauda and Ca-alginate for biosorption of Cu(II), Zn(II) and Ni(II): Kinetics and equilibrium studies. *Bioresour. Technol.* **100**(1), 186–193 (2009). 18632265 10.1016/j.biortech.2008.05.050

[81] Anielak, A. M. & Schmidt, R. Sorption of lead and cadmium cations on natural and manganese-modified zeolite. *Polish J. Environ. Stud.* **20**(1), 15–19 (2011).

[82] Huang, C. P. & Ostovic, F. B. Removal of cadmium (II) by activated carbon adsorption. *J. Environ. Eng. Div.* **104**(5), 863–878 (1978).

[83] Argun, M. E., Dursun, S., Ozdemir, C. & Karatas, M. Heavy metal adsorption by modified oak sawdust: Thermodynamics and kinetics. *J. Hazard Mater.* **141**(1), 77–85 (2007). 16879919 10.1016/j.jhazmat.2006.06.095

[84] Okolo, B. I. et al. Adsorption of lead(II) from aqueous solution using Africa elemi seed, mucuna shell and oyster shell as adsorbents and optimization using Box-Behnken design. *Appl. Water Sci.* **10**(8), 1–23 (2020).

[85] Bakatula, E. N., Richard, D. & Zagury, G. J. Determination of point zero of charge of natural organic materials. *ESPR* **25**, 7823–7833 (2018). 29294236 10.1007/s11356-017-1115-7

[86] Kragović, M., Stojmenović, M., Petrović, J., et al. Influence of Alginate Encapsulation on Point of Zero Charge (pH pzc ) and Thermodynamic Properties of the Natural and Fe(III)-Modified Zeolite. In: *Procedia Manufacturing*. Vol 32. Elsevier. 286–293 (2019).

[87] Chinnakoti, P., Chunduri, A. L. A., Vankayala, R. K., Patnaik, S. & Kamisetti, V. Enhanced fluoride adsorption by nano crystalline γ-alumina: Adsorption kinetics, isotherm modeling and thermodynamic studies. *Appl. Water Sci.* **7**(5), 2413–2423 (2017).

[88] Hallajiqomi, M. & Eisazadeh, H. Adsorption of manganese ion using polyaniline and it’s nanocomposite: Kinetics and isotherm studies. *J. Ind. Eng. Chem.* **55**, 191–197 (2017).

[89] Aksu, Z. Application of biosorption for the removal of organic pollutants: A review. *Process Biochem.* **40**(3–4), 997–1026 (2005).

[90] Ma, X. et al. Adsorption of heavy metal ions using hierarchical CaCO3–maltose meso/macroporous hybrid materials: Adsorption isotherms and kinetic studies. *J. Hazard Mater.* **209–210**, 467–477 (2012). 22326246 10.1016/j.jhazmat.2012.01.054

[91] Meena, A. K., Mishra, G. K., Rai, P. K., Rajagopal, C. & Nagar, P. N. Removal of heavy metal ions from aqueous solutions using carbon aerogel as an adsorbent. *J. Hazard Mater.* **122**(1–2), 161–170 (2005). 15878798 10.1016/j.jhazmat.2005.03.024

[92] Wierzba, S., Makuchowska-Fryc, J., Kłos, A., Ziembik, Z. & Ochędzan-Siodłak, W. Role of calcium carbonate in the process of heavy metal biosorption from solutions: Synergy of metal removal mechanisms. *Sci. Rep.* **12**(1), 1–13 (2022). 36271239 10.1038/s41598-022-22603-4 PMC9587271

[93] Ni, B. J. et al. Competitive adsorption of heavy metals in aqueous solution onto biochar derived from anaerobically digested sludge. *Chemosphere.* **219**, 351–357 (2019). 30551101 10.1016/j.chemosphere.2018.12.053

[94] Demirkiran, N. Copper adsorption by natural manganese dioxide. *Trans. Nonferrous Met. Soc. China (Eng. Ed.)* **25**(2), 647–653 (2015).

[95] Tan, I. A. W., Hameed, B. H. & Ahmad, A. L. Equilibrium and kinetic studies on basic dye adsorption by oil palm fibre activated carbon. *Chem. Eng. J.* **127**(1–3), 111–119 (2007).

[96] Hameed, B. H. & Ahmad, A. A. Batch adsorption of methylene blue from aqueous solution by garlic peel, an agricultural waste biomass. *J. Hazard Mater.* **164**(2–3), 870–875 (2009). 18838221 10.1016/j.jhazmat.2008.08.084

[97] Septhum, C., Rattanaphani, S., Bremner, J. B. & Rattanaphani, V. An adsorption study of Al(III) ions onto chitosan. *J. Hazard Mater.* **148**(1–2), 185–191 (2007). 17368930 10.1016/j.jhazmat.2007.02.024

[98] Foo, K. Y. & Hameed, B. H. Insights into the modeling of adsorption isotherm systems. *Chem. Eng. J.* **156**(1), 2–10 (2010).

[99] Boujelben, N., Bouhamed, F., Elouear, Z., Bouzid, J. & Feki, M. Removal of phosphorus ions from aqueous solutions using manganese-oxide-coated sand and brick. *Desalin. Water Treat.* **52**(10–12), 2282–2292 (2014).

[100] Hutson, N. D. & Yang, R. T. Theoretical basis for the Dubinin-Radushkevitch (D-R) adsorption isotherm equation. *Adsorption.* **3**(3), 189–195 (1997).

[101] Dada, A. O., Olalekan, A. P., Olatunya, A. & Dada, O. O. Langmuir, Freundlich, Temkin and Dubinin-Radushkevich isotherms studies of equilibrium sorption of Zn 2+ unto phosphoric acid modified rice husk. *IOSR J. Appl. Chem.* **3**(1), 38–45 (2012).

[102] Malkoc, E. & Nuhoglu, Y. Investigations of nickel(II) removal from aqueous solutions using tea factory waste. *J. Hazard Mater.* **127**(1–3), 120–128 (2005). 16125314 10.1016/j.jhazmat.2005.06.030

[103] Lagergren, S. K. About the theory of so-called adsorption of soluble substances. *Sven Vetenskapsakad Handingarl.* **24**, 1–39 (1898).

[104] Ho, Y. S. Review of second-order models for adsorption systems. *J. Hazard Mater.* **136**(3), 681–689 (2006). 16460877 10.1016/j.jhazmat.2005.12.043

[105] Itodo, A., Abdulrahman, F., Hassan, L., Maigandi, S. A. & Itodo, H. Intraparticle diffusion and intraparticulate diffusivities of herbicide on derived activated carbon. *Researcher.* **2**(2), 74–86 (2010).

[106] Kalavathy, M. H., Karthikeyan, T., Rajgopal, S. & Miranda, L. R. Kinetic and isotherm studies of Cu(II) adsorption onto H3PO 4-activated rubber wood sawdust. *J. Colloid Interface Sci.* **292**(2), 354–362 (2005). 16040040 10.1016/j.jcis.2005.05.087

[107] Weber, W. J. & Morris, J. C. Intraparticle diffusion during the sorption of surfactants onto activated carbon. *J. Sanit. Eng. Div. Am. Soc. Civ. Eng.* **89**(1), 53–61 (1963).

[108] Hamissa, A. M. B., Brouers, F., Ncibi, M. C. & Seffen, M. Kinetic modeling study on methylene blue sorption onto agave americana fibers: Fractal kinetics and regeneration studies. *Sep. Sci. Technol.* **48**(18), 2834–2842 (2014).

[109] Vadivelan, V. & Kumar, V. K. Equilibrium, kinetics, mechanism, and process design for the sorption of methylene blue onto rice husk. *J. Colloid Interface Sci.* **286**(1), 90–100 (2005). 15848406 10.1016/j.jcis.2005.01.007

